# Time-domain brain: temporal mechanisms for brain functions using time-delay nets, holographic processes, radio communications, and emergent oscillatory sequences

**DOI:** 10.3389/fncom.2025.1540532

**Published:** 2025-02-18

**Authors:** Janet M. Baker, Peter Cariani

**Affiliations:** ^1^Massachusetts Institute of Technology, Cambridge, MA, United States; ^2^Harvard Medical School, Boston, MA, United States

**Keywords:** temporal coding, time-delay neural networks, oscillations, multiplexing, holography, wave interference, radio communications, time-domain

## Abstract

Time is essential for understanding the brain. A temporal theory for realizing major brain functions (e.g., sensation, cognition, motivation, attention, memory, learning, and motor action) is proposed that uses temporal codes, time-domain neural networks, correlation-based binding processes and signal dynamics. It adopts a signal-centric perspective in which neural assemblies produce circulating and propagating characteristic temporally patterned signals for each attribute (feature). Temporal precision is essential for temporal coding and processing. The characteristic spike patterns that constitute the signals enable general-purpose, multimodal, multidimensional vectorial representations of objects, events, situations, and procedures. Signals are broadcast and interact with each other in spreading activation time-delay networks to mutually reinforce, compete, and create new composite patterns. Sequences of events are directly encoded in the relative timings of event onsets. New temporal patterns are created through nonlinear multiplicative and thresholding signal interactions, such as mixing operations found in radio communications systems and wave interference patterns. The newly created patterns then become markers for bindings of specific combinations of signals and attributes (e.g., perceptual symbols, semantic pointers, and tags for cognitive nodes). Correlation operations enable both bottom-up productions of new composite signals and top-down recovery of constituent signals. Memory operates using the same principles: nonlocal, distributed, temporally coded memory traces, signal interactions and amplifications, and content-addressable access and retrieval. A short-term temporary store is based on circulating temporal spike patterns in reverberatory, spike-timing-facilitated circuits. A long-term store is based on synaptic modifications and neural resonances that select specific delay-paths to produce temporally patterned signals. Holographic principles of nonlocal representation, storage, and retrieval can be applied to temporal patterns as well as spatial patterns. These can automatically generate pattern recognition (wavefront reconstruction) capabilities, ranging from objects to concepts, for distributed associative memory applications. The evolution of proposed neural implementations of holograph-like signal processing and associative content-addressable memory mechanisms is discussed. These can be based on temporal correlations, convolutions, simple linear and nonlinear operations, wave interference patterns, and oscillatory interactions. The proposed mechanisms preserve high resolution temporal, phase, and amplitude information. These are essential for establishing high phase coherency and determining phase relationships, for binding/coupling, synchronization, and other operations. Interacting waves can sum constructively for amplification, or destructively, for suppression, or partially. Temporal precision, phase-locking, phase-dependent coding, phase-coherence, synchrony are discussed within the context of wave interference patterns and oscillatory interactions. Sequences of mixed neural oscillations are compared with a cascade of sequential mixing stages in a single-sideband carrier suppressed (SSBCS) radio communications system model. This mechanism suggests a manner by which multiple neural oscillation bands could interact to produce new emergent information-bearing oscillation bands, as well as to abolish previously generated bands. A hypothetical example illustrates how a succession of different oscillation carriers (gamma, beta, alpha, theta, and delta) could communicate and propagate (broadcast) information sequentially through a neural hierarchy of speech and language processing stages. Based on standard signal mixing principles, each stage emergently generates the next. The sequence of oscillatory bands generated in the mixing cascade model is consistent with neurophysiological observations. This sequence corresponds to stages of speech-language processing (sound/speech detection, acoustic-phonetics, phone/clusters, syllables, words/phrases, word sequences/sentences, and concepts/understanding). The oscillatory SSBCS cascade model makes specific predictions for oscillatory band frequencies that can be empirically tested. The principles postulated here may apply broadly for local and global oscillation interactions across the cortex. Sequences of oscillatory interactions can serve many functions, e.g., to regulate the flow and interaction of bottom-up, gamma-mediated and top-down, beta-mediated neural signals, to enable cross-frequency coupling. Some specific guidelines are offered as to how the general time-domain theory might be empirically tested. Neural signals need to be sampled and analyzed with high temporal resolution, without destructive windowing or filtering. Our intent is to suggest what we think is possible, and to widen both the scope of brain theory and experimental inquiry into brain mechanisms, functions, and behaviors.

## 1 Introduction

This article outlines a general-purpose time domain framework for information processing in the brain based on temporal codes, representations, and correlation operations. In contrast to the bulk of existing theories of brain functions, which are based on complex patterns of which neural channels are activated at a given time (channel codes), here neural information processing is conceived in terms of interactions of complex patterns of spikes (temporal codes).

Its long-term goal is to understand how brains work as informational systems, to reverse-engineer brains, for both scientific and engineering ends. uapsNeurocomputational theories of information processing are neurally grounded functional models in that they attempt to explicate the kinds of functional organizations and operating principles that brains use to produce appropriate, effective behaviors. In contrast to, and complementary with, low-level detailed purely molecular, structural, dynamical, and high-level symbolic and behavioral approaches, neurocomputational theories are middle-level approaches that focus on how information processing might be realized in biological neural systems (Cariani, 2001b; Carandini, 2012; Cariani and Baker, 2022).

The time-domain perspective presented here arises from our work in both science and engineering on the perception and pattern recognition of sound, music, and speech. In the auditory domain, stimulus time structure and neural temporal codes are highly evident in the neurophysiology. Time-domain processing mechanisms appear compelling from artificial and biological signal processing perspectives. It has also been inspired by technologies of holography and radio communications.

**FIGURE 1:**
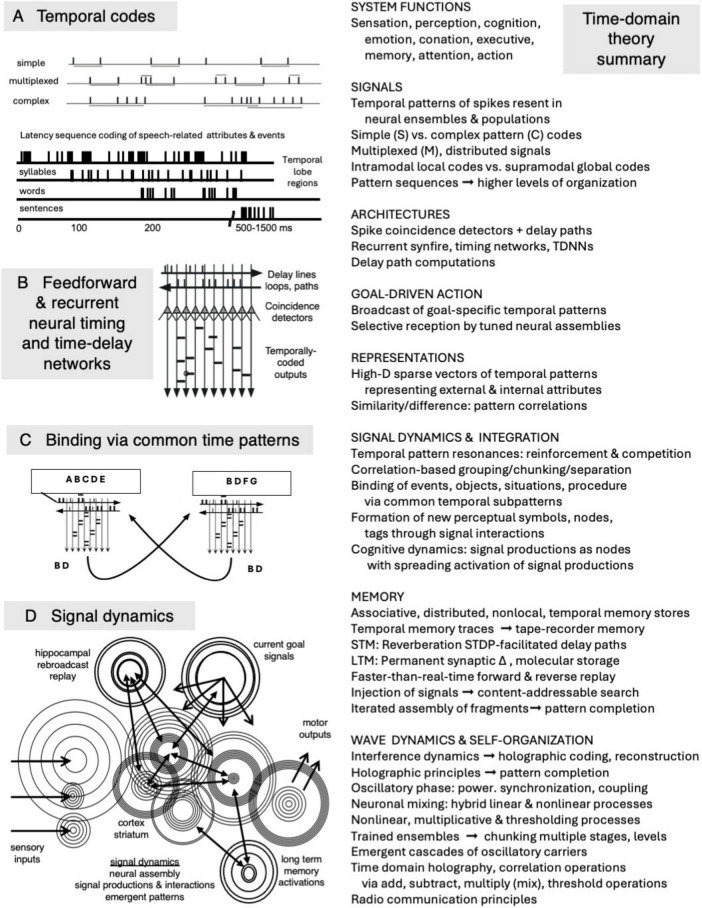
Summary of a signal-centric time-domain theory of brain function. **(Left)** Temporal codes, time-domain neural nets, correlation-based binding processes, and signal dynamics. **(Right)** Major conceptual components of the theory. Diagrams adapted from Cariani, (1997; 2015) and Cariani and Baker (2022).

The major assertions of the theory are that peripheral and multiplexed central temporal codes and neural temporal processing architectures may subserve (1) many, possibly all, informational distinctions, and central neural representations, (2) many, possibly all, and major informational functionalities of brains [see section “2 Basic brain functionalities (what is to be explained),” Table 1, e.g., sensation, perception, cognition, emotion, motivation, attention, executive functions, orchestration of action, and motor programs], (3) correlation-based mechanisms of segmentation and binding, (4) associative memory representations and operations, (5) multidimensional cognitive dynamics via spreading activations of temporally coded neural signals, (6) emergence of new signals via nonlinear and multiplicative signal interactions, and (7) temporal coding of the multidimensional contents of memory traces. We further posit (see section “4 Time-domain waveforms, signals, and systems: common signal operations, holography, radio communications, and the brain”) that time-domain representations and operations can be effected using temporal correlations, time-domain holographic-like processing, wave interference patterns, and/or oscillatory cascades. These points are also outlined in Figure 1.

**TABLE 1:** What is to be explained by a general time-domain theory of brain function.

Functionality	Functions	Temporal codes	Operations	Structures
*Bodily regulation* Maintain homeostasis Manage bodily processes	Manage circulation, respiration, digestion, metabolism, immunity	Repetitive actions and sequences regulated by central pattern generators	Set control and timing parameters for cells, tissues and organs	Hypothalamus Autonomic NS
*Modal control* Switching of global modes of operation	Wake/sleep/trance Sequencing sleep stages Stereotyped behaviors	Possible driving via temporal patterns (e.g., oscillatory modes)	Switching, maintenance, and sequencing of modes	Hypothalamus
*Sensation* Registration of events external to nervous system Registration of sensory consequences of actions	Readouts on current state of environment and body Proprioceptive feedback of muscle actions	Phase-locked and non-phase-locked temporal patterns Population statistics of temporal spike patterns	Neural encodings of sensory distinctions Encoding of stimulus time structure Similarity: correlation	Sensory receptors afferent pathways
*Perception* Organization of sensations	Perceptual organization Representations Similarity and invariance Grouping, binding Object and event formation Chunking, separations Perceptual symbols	Temporal correlation-based representations Perceptual distances based on correlation magnitudes	Object and event formation/separation via common temporal correlations Context-dependent Δ’s Attribute-grouping Temporal grouping	Afferent and efferent sensory pathways Unimodal cortex Association cortex
*Cognition* Pattern recognition Concepts Categorical reasoning Language Causal models	Pattern recognition Categorical perception Multi-D vectorial reps Compositionality Spreading activation Unsupervised and supervised causal models	New temporal patterns emitted by trained neuronal assemblies Perceptual symbols New symbols: temporal spike patterns frequencies, tags, labels	Concept mechanics: signal dynamics of high-D temporal pattern vectors Symbol formation Features → symbols Symbols → features	Cerebral cortex Distributed concept nodes: neural assemblies produce temporal patterns, signify semantic pointers
*Emotion* Emotion and mood Affective states	Readouts on current dispositions re: modes of prospective action (fight, flight, mate)	Characteristic temporal patterns associated with different affective state (e.g., Clynes, 1977)	Biases all internal processes according to emotional state (e.g., anger biases behavior)	Amygdala Cerebral cortex Autonomic NS
*Purposive control* Purposes, goals, drives, task-selection (conation)	∙ Drives, purposes ∙ Goal representations ° Simple vs. complex ° Short-/long-term ∙ Priorities, competition ∙ Evaluation/reward: goal satisfaction ∙ Steering, stop conditions	Characteristic spike patterns associated with current system goals and purposive behavioral modes that are widely broadcast	∙ Systemic broadcast of current goals ∙ Suppression of secondary goals ∙ Attentional focus: organize system for goal-appropriate actions	Orbitofrontal cortex Amygdala Other structures Goal-driven control via striatal circuits
*Executive functions* Decision-making What to do How to do it (planning) Procedural sequences	Goal prioritization Perceived affordances Action selection Anticipation: estimation of consequences Orchestration/sequencing of actions	Temporally coded task-specific signals for selective activation of goal-relevant circuits and signals	Retrieval of event sequences and rewards from memory to predict and weigh action consequences Broadcast of chosen action-sequences	Prefrontal cortex Premotor cortex Integration of high level multimodal information for action
*Attention* Selection/facilitation of specific channels and/or signals	∙ Voluntary: suppression, activation of goal-relevant channels, signals ∙ Involuntary: focus on salient, unexpected events	Signal enhancement via ∙ Amplification and attenuation of signals via ∙ Δ channel gains (gating) ∙ Top-down injections of matched signals	∙ Goal-directed circuit inhibition/disinhibition enhancement of goal-relevant signals, suppression of others	Basal ganglia Cerebral cortex Thalamus Sensory pathways Cerebellum
*Action orchestration* Action preparation: circuit activations, sequencing and timings	Priming of motor programs and circuits relevant for achieving current goals	Motor programs as temporal sequences of activations of muscles Time structure of motoric action	Task-specific disinhibition of task-relevant circuits inhibition: competing action subsystems Sequencing and timing within motor programs	Premotor cortex Basal ganglia Thalamus Cerebellum (timing)
*Timing relations*	∙ Regulation of timing of internal operations ∙ Adaptive adjustment of internal delays ∙ Time-shifting and -warping operations	Modal and supramodal temporal coding of event timings (onsets, offsets, durations, interevent delays, formation of event timelines)	Real-time adjustment of relative internal delays in perception and action Commonalities of event time structure in perception and action	Cerebellum, basal ganglia Time warping in sensory and motor planning structures
*Action* Motor action	Effecting changes in body and external environment	Temporal patterns of effector (muscle, secretory) activations	Central pattern generators Reflexes	Can be oscillatory or TDNN
*Memory* Short-term (STM): fast temporary, reverberant, echoic, visual Long-term (LTM): slower, permanent, distributed LTM mechanisms: trained neural assemblies intra-neural resonances (cellular, molecular)	∙ Memory: time-shifting mechanisms (storage and delayed recall) ∙ Past history guides present behavior ∙ Content-accessible: incoming signals activate similar traces ∙ Trained neural assemblies produce (stored) temporal traces	∙ Temporal memory traces (STM, LTM): multiplexed temporal spike patterns ∙ Storage via STM reverberation, replay and LTM consolidation ∙ Binding: local and global trace fragments bound via common time patterns Assembly of fragments	STM: reverberation in delay loops via STDP-mediated facilitation/inhibition Consolidation (STM → LTM) Hippocampal replay Time compression Trace assembly and completion (slower) Trace assembly	Cerebral cortex, hippocampus, striatum, etc. Mechanisms: STM: STDP LTM: (1) Δ synapses (2) molecular and cellular temporal resonances
*Reward* Internal feedback from consequences of action (enables supervised learning)	Evaluation of desirability of effects of actions Reinforcement of action-sequences that produce goal-satisfaction	∙ Broadcast of temporal-coded reward labels ∙ Non-temporal control signals → reinforce, suppress signals	Learning Adaptive adjustments Supervised training of neural assemblies Signal dynamics	Midline dopamine and basal ganglia circuits
*Learning* From system history, Δ internal organization to (i) better predict future (ii) better satisfy goals	Adaptive process to improve prediction, performance via reward or event correlations	Enhancement/suppression of temporally coded signals correlated with rewards or predictions	One- and multiple-shot learning: STM → hippocampal replay and consolidation → LTM (Δ neural assemblies)	Plasticity in almost all sectors of the brain

This table is intended to be a simplified overview of basic mental functionalities and their proposed temporal codes, representations, operations, and types of brain structures thought to be most directly involved.

To our knowledge, no other such comprehensive, pan-temporal theory of brain function has ever been proposed. Perhaps the closest would be theories of event timing sequences mediated by hippocampal replay and time cells (Eichenbaum, 2013; Howard et al., 2014), where timings of time cell responses and/or which time cells are activated can encode event sequences on coarse timescales, but these theories do not propose that the attributes of events, i.e., what different attributes distinguish one event from another, are also themselves temporally coded. There also exist mathematical models of representations of time that rely on temporal receptive fields of time cells (Lindeberg, 2023; Howard et al., 2024). Many functional parallels with Grossberg’s (2021) adaptive resonance theory can be drawn, except that in this time domain theory the pattern resonances involve signals consisting of temporal patterns of spikes, whereas in his theory the functional states consist of patterns of firing rates among neural channels.

We do not by any means claim that the theory is correct in all respects, but, given the present, relatively rudimentary state of brain theory, we do firmly believe that this possibility needs to be on the table for discussion, consideration, and investigation. The theory is open to empirical test by neurophysiological and neuropsychological experiments, some of which are outlined in section “5 Discussion – testing the time-domain theory.” Confirmation or dismissal of the theory will require solving the neural coding problem in central brain circuits, i.e., identifying the specific neural correlates of the specific informational contents of the attributes encoded in neural representations. Higher temporal resolutions and concerted efforts to identify temporal correlations and embedded patterns amongst spikes will be needed. Observation of nonlinear wave interactions and oscillatory cascades will require high resolution multi-region recordings and methods of analysis of neural responses that, in contrast to windowed Fourier analyses, do not smear out fine temporal structure.

Although many of the formative ideas that constitute parts of the temporal theory can be found in our earlier writings, this is the first time that we have attempted to incorporate them into a common framework. These elements began with time-domain analyses of speech (Baker, 1975), temporal coding of pitch (Cariani and Delgutte, 1996) and surveys of evidence for temporal coding of sensory information (Cariani, 1995, 2001c). Neural timing nets and time-domain correlation operations for matching common attributes of different stimuli, such as pitch, timbre, and rhythm were proposed (Cariani, 2001a; Cariani, 2002). Separating instruments and voices with different F0-pitches (auditory scene analysis and cocktail party problem) was demonstrated using correlation-based operations implemented via recurrent timing nets (Cariani, 2004). Work on the neural correlates of sequential hierarchical stages of speech and language processing has informed the cascade model presented here (Baker et al., 2011; Chan et al., 2011). Our previous paper (Cariani, 2022 Cariani and Baker, 2022) focused on possible types of central temporal codes, processing architectures, and connections to wave dynamics, holography, and oscillatory cascades, but did not attempt to relate these in any systematic way to the gamut of major brain functions. The present paper attempts to account for all these functions within a unified temporal representation and processing rubric. A strength of the study is that it offers an entirely new perspective on brain function. A limitation is that the theory is in early stages of development such that many parts of it have not been formalized. As best we know, this theory is not contradicted by available evidence.

What is novel about this theory? Time-domain approaches break with longstanding assumptions that regard brains as connectionist neural networks. In mainstream theories, neural functional states are described in terms of levels of activation (e.g., firing rates) amongst a set of elements (i.e., individual neurons, ensembles, and populations). In contrast, the time-domain conception regards brains as temporal correlation machines in which information is temporally coded. It begins with the alternative assumption that the signals of the system themselves (mostly) consist of temporal patterns of spikes. In the standard view, the functional informational states of brains are large vectors that represent across-neuron profiles of firing rates. These vectors are also called rate-place patterns, place being a neuron’s position within the network, its network connectivity. However, it is also possible that these functional states are instead vectors consisting of different temporal patterns of spikes that circulate in network delay paths.

Rather than a *channel-centric* theory of neuronal representations and information processing architectures, an alternative, time-domain, *signal-centric* theory is proposed. In the signal-centric view, different sets of neural signal productions switch and organize the action of the system to produce different behaviors. This involves a paradigmatic change of perspective in which most of the action is in the neural signals and their interactions rather than in which subsets of neurons happen to be most active.

Figure 1 illustrates the basic elements of the theory: a neural coding framework that includes simple, complex, multiplexed, local and global temporal codes, time-domain neural processing architectures, grouping operations based on common temporal patterns, and signal dynamics that support signal competition and mutual reinforcement, spreading activation, and content-accessible activation of memory traces. The theory draws on mathematical and technological principles of correlation operations, holography, and radio communications technology for potential means by which distributed, associative, content-addressable memory storage and access might be implemented using time-domain neural signals and mechanisms.

In a nutshell, temporally patterned spike trains arriving from sensory surfaces interact with each other and with those produced by existing, trained central neural assemblies to form regenerated sets of signals that circulate in reverberating short-term memory delay paths, including echoic and working memory. These signals in turn interact with those associated with current goals, affective states, and long-term memories to prepare the system for appropriate action and to orchestrate corresponding motoric temporal sequences.

The temporal theory requires a conceptual shift from digital to analog modes of signal representation and processing. Historically the dominant theories of brain function have assumed channel-based neural architectures that rely on inputs from arrays of dedicated tuned, feature-sensitive neural elements (i.e., “feature detectors”). These theories paralleled the sequential-hierarchical organization of information processing in digital electronic computers. Patterns of activation amongst arrays of these neural feature detectors or filters were traditionally assumed to be the default mode of representation in the central nervous system. Additionally, it was assumed that each neural element was sensitive to one attribute (feature), i.e., one neuron and one attribute. Their output signals were assumed to be one dimensional, scalar time-series signals in the form of moving averages of firing rates or spiking probabilities over tens to hundreds of milliseconds. Here for neurons with very low firing rates, a single spike can be significant. The basic assumption of coding based on moving time-averaged rates ruled out of hand any concurrent or interleaved multiplexing of different types of information. Ensembles of these scalar neural signals were thought to be subsequently processed using switchboards with highly specific interneural connectivities, synaptic weightings, and tunings (John, 1972; Eichenbaum, 2018).

Instead, the temporal theory proposes time-domain representations and operations based on temporal correlations of spikes. Major central neurocomputational structures (cerebral cortex, striatum, thalamus, hippocampus, and cerebellum) are re-envisioned in terms of arrays of neural delays and coincidence detectors that process temporally structured spike patterns, e.g., temporal correlation machines.

As used here, *time-delay networks* encompass two types of neural networks: those that dynamically self-organize to form temporary reverberating short-term representations and expectancies (e.g., synfire chains, neural timing nets, and wave interference networks), and those that have been organized through many repeated rewarded and/or salient unrewarded input patterns to form quasi-permanent, selective neural assemblies (e.g., classical time-delay neural networks, TDNNs). In the temporal view, dynamic and permanent changes in synaptic efficacies enable adaptive facilitation and selection of delay paths, permitting these two modes of self-organization and learning. Whereas both modes are based on spike temporal correlations, the dynamic changes are mediated by short-term spike timing dependent facilitations, whereas more permanent changes are mediated by long-term synaptic and molecular modifications.

The reverberatory networks that subserve short-term memory are erasable *tabula rasas* that have no permanent structure, making them capable of temporarily holding any contents, including novel input patterns that the system has never before encountered. In contrast, the permanent neural assemblies are tuned through accumulated experience to selectively respond to and produce particular temporal, spatial, or spatiotemporal patterns of spikes. In neural network terms, the reverberating networks can be regarded in terms of (unsupervised) synfire chains/cycles, neural timing nets, and oscillator networks, whereas the more permanent, long-term neural assemblies can be regarded in terms of (supervised and unsupervised) trainable TDNNs, oscillator networks, and central temporal pattern generators. All of these alternative network types are potentially capable of analyzing and producing temporally patterned neural spike train signals.

In this time-domain theory, reverberatory networks support labile, temporary short-term memory, whereas permanent synaptic modifications in neural assemblies, perhaps aided by molecular mechanisms (Cariani, 2017), support non-labile long-term memory. For both short- and long-term memory, the time-domain theory postulates a “tape recorder-like” process in which memory traces consist of temporal patterns of pulses. Memories associated with particular objects, events, situations, and episodes may reside in collections of related local and global temporal memory fragments that reside in different neural populations. The fragments are assembled and bound through an iterative, spreading activation process based on shared temporal subpatterns. This mode of distributed, content-accessible storage bears many similarities to wave interference dynamics, radio communications and holography (see section “4 Time-domain waveforms, signals, and systems: common signal operations, holography, radio communications, and the brain”), albeit in their less common correlation-based and time-domain variants.

For the most part, this present theory is framed in terms of temporal codes and neural timing nets, but hybrid combinations of temporal- and channel-based networks (e.g., TDNNs) are by no means ruled out of hand. The temporal approach questions many unexamined, default coding assumptions. Because stimulus- and situation-specific differences in firing rates amongst neurons that are widely observed may co-occur with differences in spike latencies, orders-of-firings, and temporal patternings, it is important to disambiguate these alternative coding possibilities by examining how well each candidate code predicts some specific function, be it a percept, emotional or cognitive state or overt behavior. Activation of specific sets of neurons can trigger the subsequent production of temporal patterns of spikes downstream (as in central pattern generators), or vice-versa. In all cases, correlations and causal linkages between patterns of firing rates and spike correlations and behavioral functions need to be carefully explored. Many of these neural coding questions may be eventually resolved through fine-grained spike train analyses and targeted interventions, such as electrical, magnetic, and optogenetic driving, of individual neurons and local ensembles.

Formulating and proposing a new, tentative theory based on time-domain signals and their dynamics is a daunting task. Such a theory needs to be consistent with available experimental neuroanatomical, neurophysiological, and neuropsychological evidence. In the past, most neuroscience textbooks have assumed that temporal coding is limited to sensory peripheries in specific modalities such as audition, mechanoreception, and electroreception, where phase-locked spikes are ubiquitous and obvious and spike timing information can reliably predict percepts with high precision (Cariani, 2001c; Cariani and Baker, 2025, under review)^1^. However, if one looks deeper into the literature, evidence for temporal coding can be found for virtually all modalities, including vision, the chemical senses, and pain (Perkell and Bullock, 1968; Cariani, 2001c). Because stimulus-related spike timing information is less obvious as one ascends sensory pathways, most theorists have adopted as a default assumption that temporal codes must be converted to channel-codes by the time spikes coursing through these pathways reach the cortex. However, the present state of understanding of neural coding in most central stations (e.g., cerebral cortex, striatum, hippocampus, and cerebellum) is still quite rudimentary, with many unresolved questions and confounds. Neural coding in these places has proven to be an extremely difficult problem to solve, such that there are few examples where even basic sensory attributes, such as shape, color, texture, pitch, timbre, phonetic distinctions, smell, and taste can now be reliably predicted with any high precision from cortical or hippocampal spike train data. Temporal patterns of spikes may exist interleaved with other patterns or as temporal correlations across neurons. These are forms of order that are notoriously hard to detect, unless concerted directed efforts are made to search for them using well-defined, interpretable stimuli. Even with the massive neural datasets now available, it is still difficult to rule out spike correlation codes out of hand.

There are many potential advantages of central time-domain neural architectures that use temporal codes in whole or in part, that drive interest in developing a general time-domain theory. These include the precision, robustness, and invariance of temporal coding of sensory distinctions; simplicity of encoding temporal relations between events, a common coding framework for all types of primitive features; combinations of temporal patterns for multidimensional representations of objects, features, situations, and internal procedures; perceptual grouping by common temporal subpatterns; signal multiplexing in spike trains of individual neurons and ensembles; multivalent neurons that are sensitive to multiple stimulus types and attributes; selective reception of specific temporal patterns; broadcast-based communication and control; content-addressable memory; nonlocal and distributed temporal patterns; temporal memory traces, tape-recorder-like memory storage and readout, compositionality; creation of new temporal patterns through nonlinear thresholding and multiplicative operations; and wave interference and holographic-like distributed storage in the time domain.

A general time-domain theory proposes that temporally coded neural spike patterns carry specific perceptual, cognitive, emotional, motivational, mnemonic, and motoric distinctions. Behavior is produced through competitive and cooperative signal dynamics in which the various signals interact to mutually reinforce, suppress, separate, or combine. In terms of metaphors, brains appear to be more like analog radio communication-control systems and holograms than discrete switchboards and conventional connectionist networks.

The paper first outlines the basic informational functionalities that need to be accounted for in any general neurocomputational theory of brain function [see section “2 Basic brain functionalities (what is to be explained)]. Then neural architectures, representations, and operations needed to carry out these functionalities – temporal neural codes, time-domain neural networks, multidimensional representations, binding processes, and signal dynamics are discussed (see section “3 Proposed time-domain operations and mechanisms”). Prospective functional principles taken from wave dynamics, holography, and radio communications systems are then considered (see section “4 Time-domain waveforms, signals, and systems: common signal operations, holography, radio communications, and the brain”).

A great deal of evidence from psychology and neuroscience strongly suggests to us that neural systems operate on multiplexed signals in the time domain. However, this theory is in initial stages of formulation, and far from complete, so this presentation should be taken as only a rough sketch rather than a fleshed-out, finished neurocomputational model. The final section (see section “5 Discussion – testing the time-domain theory”) discusses how the theory might be tested.

## 2 Basic brain functionalities (what is to be explained)

**FIGURE 2:**
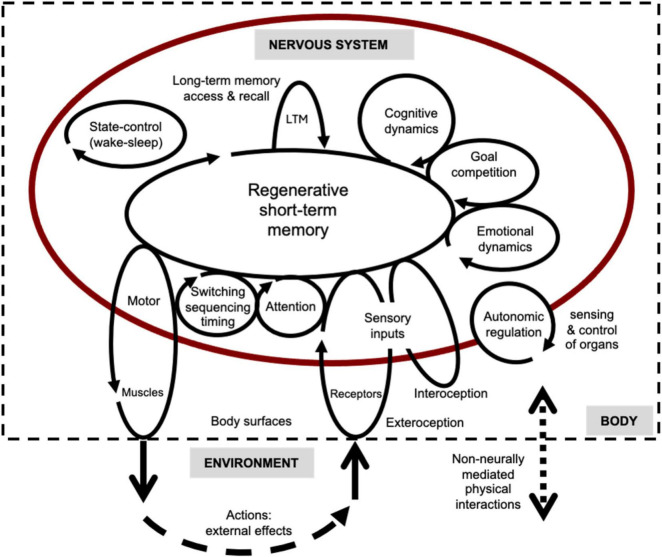
Functional schematic of brains as networks of circular-causal processes. Process loops consisting of sets of circulating neural signals that link three realms of environment, body, and nervous system. Each process loop in the diagram is related to a different basic functionality (shown) and its corresponding underlying neural circuits (not shown). Signals build-up, compete, persist, and decay in the circuits. This is an updated version of an earlier figure.

A general theory of how brains work seeks to explain how all the various basic, essential modes of internal information processing – mind-brain functionalities – can be realized by specific types of neural networks operating on specific types of neural signals. The schematic of Figure 2 depicts most of these functionalities in terms of recurrent internal processes embedded in goal-directed percept-coordination-action loops. These sets of loops are involved with interactions of the nervous system with the rest of the body (interoception and autonomic functions) and its external environment (exteroception and action). The functionalities are intended as a general framework for explaining animal behavior.

The basic functionalities of Figure 1 are listed in more detail in Table 1 and in following sections. These include:

• *Regulation of bodily functions* – Neural management of cells, tissues, and organs for maintaining system integrity (homeostasis).
• *Modal control* – Switching of system-wide functional states, such as wake-sleep cycles, levels of arousal, hypnotic and trance states, sleep stages, and stereotyped behavioral modes.
• *Sensation* – Interaction of sensory organs with the body (interoception) and world external to it (exteroception) to provide information regarding their current states.
• *Perception* – Organized sensations, neural representations, invariances/similarities/differences, object and event formation/grouping/separation, pre-attentive non-acquired, built-in expectancies, bottom-up depending on current and recent sensory signals, and modulated by top-down attentional, emotional, cognitive, and mnemonic contexts.
• *Cognition* – Pattern recognition; categorical perception; conceptual representations, operations, and dynamics; language understanding and production; and internal models and acquired expectancies.
• *Emotion* – Readout of the global state of the organism (e.g., anger, fear, interest, and affection) associated with current dominant mode of prospective action, e.g., fight, flee, explore, play, approach, and avoid/hide. Emotion-related neural signals, hypothesized to be temporally coded, are broadcast widely, biasing behavioral response modes and priming circuits related to all the other functionalities.
• *Purposes* – Internal systemic goals (conation and motivation) that organize neural circuits to steer behavior so as to bring about attainment (satisfaction) of current goal states. Goal signals are postulated to be temporally coded and broadcast widely.
• *Executive functions* – Deciding which individual goals to act upon, and in what order, and choosing which actions to take to attain them. These functions include decision-making and action selection: recognizing affordances (goals that are attainable within current situations), weighing priorities and urgencies of competing goals, assessing their respective current likelihoods of attainment, and planning appropriate action sequences. Through competitive, mutually suppressive, winner-take-all processes one goal or set of related goals tends to drive behavior at any given moment. Neural signals for different goals compete (see section “3.5 Signal dynamics: mutual reinforcement, competition, and spreading activation”) with the emergent, currently dominant goal-signals being widely broadcast throughout the brain, where they facilitate task-relevant attention and action preparation.
• *Attention* – Selective enhancement of specific neural channels, circuits, and/or signals. Attentional processes amplify signals relevant to current goals (voluntary attention) and/or to unexpected, perceptually salient events (involuntary attention). Signal-to-noise ratios of relevant signals can be improved by suppressing irrelevant channels and signals. Attentional facilitation for detecting particular patterns can also be realized by injecting matched signals from memory that are similar to the attributes of the object being sought (e.g., by imagining the object’s appearance). These top-down signals then reinforce similar bottom-up signals coming from sensory pathways.
• *Action preparation and orchestration* – Priming of motor programs and circuits relevant for achieving current goals and suppression of competing programs. Implementing sequencing and timing of procedures within motor programs.
• *Action* – Activity by effectors (muscles and secretory organs) that alter the states of the rest of the body and the external world.
• *Evaluative feedback* – Following an action or action sequence, positive and negative reward signals related to its efficacy in satisfying specific goals are fed back to decision processes. Reward signals within dopamine-mediated circuits encode an evaluation of success or failure that is then widely broadcast to the rest of the brain. These signals facilitate retention of recent rewarded action sequences in short- and long-term memory and promote adaptive reorganization of neural circuits to bias future action sequences to favor successful ones (reinforcement learning).
• *Memory* – Storage and later retrieval of representations of internal neural events for guiding future behavior. Internal neural events can be related to unrewarded recurring perceptual patterns (causal models, expectancies, and unsupervised learning) or to rewarded percept-action sequencies (supervised and reinforcement learning).
• *Learning* – Adaptive adjustment of neural circuits that modify internal functional states, procedures, and external behaviors on the basis of previous rewarded and unrewarded histories of predictions and actions. In their broadest senses, learning can be regarded as a kind of memory, and memory as a kind of learning.

The time-domain theory seeks to explain all these basic, essential psychological, mental functionalities [see section “2 Basic brain functionalities (what is to be explained)] in terms of neural temporal processing architectures (see section “3.2 Neural architectures for temporal processing”) that operate on temporally coded signals (see section “3.2”).

Omitted from explicit mention in the schematic and the table are dysfunctional, pathological states (modes of system failure), and social-psychological dynamics. So too is conscious awareness, which may be an inherent aspect of organized neuronal activity in minds and brains rather than an evolved, naturally selected function. In accord with global neural workspace theories (e.g., Dehaene, 2014), the temporal theory posits that the basic state of awareness depends on coherent regeneration of neural signals in global and local recurrent loops. Because signals determine the functional organization of the system, this is a form of organizational closure and provisional stability that constitutes the most fundamental requisite for any conscious, experiential state, i.e., an essential component of the “neural correlates of consciousness” (NCCs). The multi-modal, phenomenal contents of awareness are held to be the sets of particular neural signals currently circulating in global and local circuits. These signals constitute the “neural correlates of (the various possible) contents of consciousness” (NCCCs).

## 3 Proposed time-domain operations and mechanisms

Brains function to regulate internal bodily processes and to coordinate perception and action in a manner that enhances survival. Brains are purposive control systems that analyze neural activity patterns associated with sensory, emotional, motivational, cognitive, and motoric states to produce highly structured neural activity patterns that subserve complex behaviors. They are representational systems that enable complex combinations of attributes to be held in short- and long-term memory stores. They are internal communications systems that allow information to be widely broadcast and selectively received.

To understand fully how brains work as informational systems, one must understand the nature of the signals of the system, the neural architectures that produce and process them, the representational frameworks that encode the attributes and consequences of objects, events, situations, and actions, and the mnemonic processes that permit stored records of past experience to inform prospective action.

### 3.1 Neural codes: signals of the system

Neural codes are the signals of the system, i.e., those aspects of neural activity that carry informational distinctions that are relevant to the functioning of the system and its subsequent survival. As with the genetic code, our working hypothesis is that there is one common neural coding framework within which all types of information related to all brain functions can be encoded, transmitted, stored, retrieved, and used in service of preparation and action. This universal framework needs to be able to account for all the many, diverse distinctions that the biological brains of a given species can make. It must have dimensional structure and informational capacity to support the many neural activity patterns that distinguish different sensory modalities, cognitive categories, emotional and motivational states, and possible motoric actions.

#### 3.1.1 What is encoded?

What kinds of specific distinctions are encoded in this framework? A distinction here is any difference in neural activity that switches internal functional states or resultant behaviors. These include all of the sensed properties (“attributes”) of external objects, events, and situations as well as those related to internal neural states (internal attributes, such as those related to current goals and rewards, thoughts, emotions, and internal procedures related to actions). Only a small subset of the distinctions made by the nervous system are ever consciously experienced.

#### 3.1.2 Temporal codes

Temporal codes are neural codes that convey informational distinctions through simple and complex patterns of spike timings. Temporal coding is found widely throughout sensory systems, and it has the potential to be a universal strategy for encoding most, if not all, types of sensory distinctions (Cariani, 1995, 1999, 2001c).

Temporal codes are fundamentally correlation-based codes in that they depend on the joint occurrences of spiking events that have particular timing relations in the form of time durations between events. They are mixed digital-analog signals in that the signals of the system consist of discrete pulsatile events (action potentials and spikes), but the various durations between spiking events can take on continuous sets of values, as in an analog signal.

Temporal codes can be further subdivided into temporal pattern vs. relative spike timing codes. Temporal pattern codes rely on specific temporal patterns of spikes within the same neural channels such that they produce auto-correlation-like representations (e.g., for pitch, tempo, and echo delay) whereas relative spike timings across different channels produce temporal cross-correlation-like representations (e.g., for localization of sound direction, location of a stimulus on a body surface, and visual motion detection).

At the lowest level of neural coding, interspike interval codes are the simplest temporal pattern codes. Here the time durations between spikes convey information (Figure 1A). These can involve durations between pairs of consecutive spikes (first-order intervals) or non-consecutive spikes (all-order intervals). Higher order interval sequence codes can involve characteristic temporal sequences or combinations involving many more than two spikes. Extended temporal patterns can also encode sequences of events, including relative event timings and, potentially, all of each event’s attributes. Such a coding scheme was proposed in Cariani and Baker (2022), and is schematized in the bottom-most set of spike trains in Figure 1A.

Temporal codes may be at least as fundamental and phylogenetically primitive as channel-based codes. Although rate-channel coding, with its tuned elements and selective receptive fields, has been by far the dominant assumption in neuroscience, evidence for temporal coding exists throughout the nervous system, often in quite unexpected places, such as in vision, pain, and the chemical senses.

Temporal codes and channel codes are by no means mutually exclusive – they can exist in combination as hybrid and joint codes (Cariani, 1999, neural coding taxonomy). For example, the Jeffress time-delay neural net model of binaural direction relies on channel-specific encodings of interaural time differences that combine a spike relative latency code with a channel-coded output. Activations of specific neuronal channels can produce temporally patterned outputs, e.g., via TDNNs, temporal pattern generators, oscillator networks, and rhythm assimilating neurons (Morrell, 1967).

#### 3.1.3 Types of temporal codes: phase-locked and non-phase-locked sensory systems

Temporal codes in sensory systems can also be classified in terms of their relationship to the structure of the stimuli that they encode, whether their time structure comes from time-locking to a stimulus or from stimulus-triggered responses whose time structure is generated by characteristic neuronal interactions.

• *Stimulus-locked (“phase-locked”) codes*. Virtually all neurons fire preferentially in response to positive phases of stimuli that drive their excitatory inputs. In many sensory modalities, such as audition, vision, mechanoreception (touch), and electroreception, the timings of spikes produced by primary sensory neurons follow the time-structures of their respective stimuli. These stimulus-driven time-locked spikings, widely known as “phase-locking,” reflect the internal time structure of stimuli. Stimuli impress their time structure on that of the spike trains to produce iconic neural time-domain representations that resemble stimulus waveforms.

The temporal structures of the spike trains produced contain information directly related to specific perceptual attributes. In the first stages of neural representation of sounds in the auditory system, as a direct consequence of phase-locking, all-order interspike interval codes provide autocorrelation-like temporal representations of acoustic stimuli, up to the frequency limits of phase-locking (effectively up to ∼ 4 kHz). These provide robust and precise representations of perceptual qualities related to stimulus periodicities (pitch and rhythm) and low- and mid-frequency spectra (aspects of timbre, such as vowel quality) (Cariani, 1999). Analogously, on body surfaces interval codes enable flutter-vibration discrimination and relative spike timings enable localizations in tactile perception. For transient stimulus patterns (onset amplitude, frequency, and phase dynamics of sounds), spatiotemporal patterns of spike timings across characteristic frequency (CF) channels can provide cross-correlation-like representations of instrument timbres and consonant phonetic categories.

Usable phase-locking extends at least to ∼4 kHz, the highest note on a piano, in most human listeners enabling temporal representation of many attributes related to speech, music, and environmental sounds: periodicity (pitch), power spectrum (timbre and vowels), sound direction and distance (echo delay echolocation). In the auditory periphery, interspike interval representations of pitch and timbre are highly level-invariant with precisions that improve with higher sound levels, like perception but unlike the behavior of rate-place codes (Cariani, 1999; Heinz et al., 2001), such as periodicity (auditory pitch and cutaneous flutter-vibration frequency), spectrum (vowel timbres), stimulus direction (auditory and cutaneous localization, and electroreception) and distance (echolocation).

In vision, spikes phase-lock to temporal modulations of luminance as moving sinusoidal gratings travel across receptive fields, providing a precise, interspike interval-based representation of spatial frequency (Cariani, 2004) and motion (Reichardt, 1961). Many other parallels exist between auditory and visual systems: coarse temporal modulation tunings of neural elements, autocorrelation-like representations of timbre and texture, perception of missing spatial and temporal fundamentals (Reitboeck et al., 1988; Ando, 2009), as well as analogous scene analysis principles. Analogous autocorrelation and cross-correlation models for visual form based on spatial intervals (Uttal, 1975, 1988) and spatial correlation patterns (Kabrisky, 1966), respectively have also been proposed, but these rely on spatial rate-place patterns rather than spatial patterns of temporally correlated phase-locked spikes, as in Reichardt’s (1961) motion detectors or the texture scanning model of Reitboeck et al. (1988).

• Stimulus-triggered temporal codes for stimulus qualities are possible even in the absence of phase-locking of receptors. In sensory systems, such as the chemical senses and color vision, where there is no time locking of spikes to the stimulus quality being encoded, an adequate stimulus may generate characteristic temporal patterns of neuronal response in early sensory pathways that does not mimic the time structure of the stimulus quality itself (e.g., Kozak and Reitboeck, 1974; Di Lorenzo et al., 2009).

#### 3.1.4 Population-based temporal codes

Codes and representations can involve patterns of neural activity in spike trains of individual neurons or be distributed across ensembles, sub-populations, and populations. Purely temporal codes with high reliability and precision can exist at the level of whole neural populations. Codes based on temporal patterns of spikes can exist within spike trains of single neurons, within spike volley patterns produced by neural ensembles, and within the statistics of temporal correlated spikes within neural populations.

A strong example of a population-wide temporal representation for pitch and timbre exists in the mass statistics of interspike intervals (“population-interval distributions”) taken across the whole population of tens of thousands of auditory nerve fibers (Cariani and Delgutte, 1996; Cariani, 1999; Cariani, 2019). Here almost every neuron’s response carries some partial information regarding acoustic attributes, including pitch, timbre, and loudness such that the information is widely distributed across this population. In this representation, the identities of the neural channels that produce the respective temporal intervals are not needed, such that one can discard all cochlear place information and still have highly precise, robust, level-invariant representations of these attributes (e.g., F0-pitches and timbres of different single and double vowels; Cariani, 1995; Palmer, 1990). Being based on interspike intervals within individual neurons, precise synchronization of spike timing across neurons in the population is also not required. Here is an existence proof that temporal, non-place, asynchronous, and population-wide neural representations are possible.

This strong example from our own experience led us to believe that similar kinds of population-wide temporal codes might exist in more central stations and that, in conjunction with temporal correlation operations, these representations could form the basis for a comprehensive neural coding framework.

#### 3.1.5 Local and global codes

Such a framework needs to handle all kinds of distinctions, from those involving but a single-dimension, e.g., loudness or brightness, to those involving a few dimensions (color, texture, timbre, odor, and taste) to complex multidimensional spaces (e.g., multiple attributes of objects and semantics of words; see section “5 Discussion – testing the time-domain theory”). In order to handle the full, multidimensional, multimodal encoding of situations, objects, events, and procedures codes on both local and global levels may be needed. For example, many unimodal perceptual and distinctions could be carried by local codes that operate mostly within unimodal neural populations (e.g., auditory or visual cortex). Supramodal distinctions (e.g., timing and rhythmic patterns of events) could be conveyed global codes that span many brain regions. If combinations of unimodal distinctions are frequently encountered and/or significantly rewarded, then characteristic patterns arising from their signal interactions may also propagate to global circuits. The time-domain theory posits that common supramodal temporal patterns that are present to some degree in all global and local circuits bind the various types of intramodal patterns together. In this manner, not all types of information for representing complex items with multimodal attributes need be present in any one brain region.

#### 3.1.6 Finding temporal codes in neural activity patterns

A critical assumption of the time-domain theory is that many, possibly all, and informational distinctions are conveyed by temporal codes. Testing the theory therefore requires positively identifying the neural codes that subserve representations of specific attributes (e.g., attributes related to percepts such as pitch, loudness, apparent direction, shape, color, texture, apparent location, smell, taste, as well as those related to emotional, motivational, and cognitive states). Positive demonstration that a particular candidate neural code is actually used by the system entails showing a causal linkage between observed neural encodings and behavioral functions.

Although a great deal of evidence exists for temporal codes in neurons that are proximal to sensory and motor surfaces, the nature of the neural coding in central stations, e.g., cerebral cortex, striatum, cerebellum, hippocampus, and other limbic system structures, remains mostly an enigma. The further one ventures from these surfaces to address neural coding of cognitive, affective, and conative distinctions, the more these distinctions depend on other, unobserved, ill-defined, and ill-controlled internal states, and the more difficult it is to clearly identify their precise neural correlates. When we read reports of observed neuronal responses in different brain regions, we ask ourselves what the same analytical methods applied to the auditory nerve would tell us – would they be able to detect a temporal code of this sort?

Possible reasons for this difficulty are (1) that each neuron may be participating in multiple neural assemblies (they are multivalent elements, not unitary feature detectors), (2) different types of information may be multiplexed in the same spike trains, and/or (3) temporal patterns encoding informational distinctions might be distributed across neurons in volley patterns.

Reliance on experimental methods that cannot observe spike trains in individual units and analytical methods that overlook spike temporal correlations within and across single units will not detect these kinds of possibilities. Although observables, such as averaged gross electrical and magnetic responses (EEG and MEG) are useful in capturing temporally correlated activity within neuronal populations, fine timing patterns on millisecond time scales are often obscured by low-pass filtering of signals and the ways that signals are averaged.

There is always more timing information available in individual neurons than in pooled, population-wide responses. If different parts of a neural population respond with different latencies, the fine, millisecond-scale temporal structure of those responses can be smeared out and obscured. In the auditory nerve statistically significant phase-locking to 5 kHz and beyond can be observed. However, if one combines spike trains of fibers of all characteristic frequencies to form a population peristimulus histogram, due to cochlear delays, all response periodicities above roughly 200 Hz are obliterated. Even if high sampling rates above several kilohertz are used, EEG and MEG reflect only the temporally synchronized components of population responses, such that observed frequency limits of neural responses may drastically underestimate the existence of fine timing information.

Enormous progress has been achieved in identifying neural correlates of memory storage and retrieval processes, salient examples being hippocampal place-phase-codes, place and time cells, hippocampal replay/pattern completion, and the roles of population- and sub-population-wide oscillations. However, the full problem of the engram – how the various specific attributes of remembered specific objects, events, and situations are encoded in neural memory traces in short- and long-term memory – has yet to be elucidated.

### 3.2 Neural architectures for temporal processing

Brains analyze incoming sensory patterns to formulate, orchestrate, and implement appropriate actions. In order to formulate a general time-domain theory of brain function, neural networks must be reimagined as temporal correlation machines that perform time-domain operations on temporal patterns of spikes. The same central neurocomputational structures and operations are used to perform each of the various functions in Table 1, i.e., to analyze incoming sensory information, to decide what to do in terms of current goals, and to orchestrate and implement appropriate actions.

#### 3.2.1 Time-domain neural architectures

Different types of neural processing elements, operations, and architectures are required for the different types of neural codes they use for inputs, internal operations, and outputs. Taxonomies of neural networks can be formulated on the basis of what kinds of neural signals they use and what constitutes their functional states (Cariani, 2001a,c). Time-domain architectures in which spike timings play crucial roles can be contrasted with traditional “channel-domain” networks whose signals are average spike rates and whose states are characterized in terms of profiles of channel activations. These are typically rate-place activation patterns, be they dense or sparse and/or involving substantial changes in firing rates or single spiking events. Mixed time-, frequency-, and/or channel-domain neural signals and processing architectures cannot be ruled out.

The general-purpose time-domain architecture that is envisioned here has many properties in common with synfire networks (Abeles, 1990; Abeles, 2003; Abeles et al., 2004; Hayon et al., 2005), wave interference networks (Beurle, 1956; Heinz, 2010; Keller et al., 2024), neural timing nets (Cariani, 2001d; Cariani, 2004), and time-delay neural nets (TDNNs) (Jeffress, 1948; Licklider, 1951; Wang, 1995).

As with synfire chains and cycles, it consists of feedforward and recurrent networks of delay lines and coincidence detectors. As with synfire chains and wave interference networks, due to the spatial organization of delay lines, spatiotemporal patterns of spikes propagate spatially through neural populations. Patterns of interactions between waves of spikes can potentially support holograph-like distributed representations and memory traces (see section “4.2 Holography and some holography background”).

As with neural timing nets, but different from synfire networks and TDNNs, virtually all signals are also assumed to be temporally coded. TDNNs, broadly defined, are spiking neural networks that have both coincidence detectors and rate integrator elements (coincidence counters) with adjustable, stable interconnection weights and interneural delays. They can convert incoming temporal spike patterns to outgoing rate-place patterns to analyze spike temporal structure or convert incoming rate-place patterns to temporally patterned outputs as in central pattern generators.

As with TDNNs the time-domain network also has adjustable interconnection weights that can change either dynamically through spike-timing-dependent plasticity (STDP; Markram et al., 2012) or through more stable, permanent changes in synaptic efficacy. Short term memory, including echoic and working memory, is assumed to be reverberatory and dependent on STDP-mediated synaptic facilitations and depressions that are driven by spike timing correlations at synapses. Long term-memory is assumed to be mediated by more permanent synaptic changes and perhaps also by other molecular and cellular mechanisms as well that support formation of stable neural assemblies.

In time-domain networks any spatiotemporal pattern of spikes can be detected and/or produced by appropriate combinations of interconnection weights and delays. Specific interneural delays can be selected by adaptively modifying synaptic efficacies, so as to permit formation of quasi-permanent trained neural assemblies. Although these interneural delays are normally conceptualized at the level of whole neurons, there can exist slightly different delays for different synapses on the same neuron. Neurons with elaborate dendritic trees, such as cortical and hippocampal pyramidal cells, may be better regarded as trees of synapses and coincidence elements in which small numbers of well-timed spikes (spatiotemporal patterns) can trigger action potentials (Beniaguev et al., 2021). Such neural elements can participate in multiple neural assemblies by producing interleaved spikes associated with different sets of inputs (Abeles, 2009). These kinds of elements might explain why pyramidal cells with thousands of inputs have such irregular firing patterns.

Because characteristic mono- and multi-synaptic delays exist between every pair of neurons, every change in the efficacy of a given synapse produces a change in the interneural delay paths, and vice versa. Thus, if specific delays can be adaptively modified (MacKay, 1962; Gutig and Sompolinsky, 2006), then particular combinations of synaptic inputs can be arranged so as to arrive simultaneously at spike initiation points, sensitizing the neuron to those combinations.

#### 3.2.2 Neural delays and spike coincidence mechanisms

Time-domain architectures appear to be consistent with many widely observed characteristics of individual neurons. The kinds of networks that are needed to analyze and produce temporal pulse codes require neural delays and spike coincidence detectors that have relatively precise temporal resolutions. Neural delays offset time durations between spiking events, while elements with narrow duration spike coincidence windows permit spike intervals of different durations to be discriminated.

Neural time delays are ubiquitous in brains. They can be produced within individual neurons or groups of neurons. Well known neural delays include synaptic, dendritic, and axonal transmission times, time-to-threshold integration times, recovery time courses (superexcitable phases and oscillatory resonances) and post-hyperpolarization rebound times (timings of “anode break excitations”). All of these delays are potential loci for plasticity and history-dependent adaptive tunings. Other possible neural delay mechanisms that could potentially support permanent storage of temporal patterns include neuroglial-mediated processes, microtubular transmissions, and molecular storage mechanisms.

By far the most widely appreciated neural delays are “tapped delay lines,” which use axonal conduction delays, branchpoints, and collaterals of axons spatially distributed along their lengths to provide systematic sets of successive delays. Sets of tapped delay lines are found widely in many canonical central structures: in cerebral cortex, hippocampus, and cerebellum. Cell types include pyramidal and granule cells as well as binaural bipolar cells of the auditory brainstem. It is not surprising then that these major canonical neurocomputational structures can be modeled as time-delay processing architectures.

These mechanisms provide for a rich set of delays. Conduction delays in myelinated and unmyelinated axons of individual neurons span several orders of magnitude, dependent on axon lengths and conduction velocities. The most numerous cell type in the brain is the unmyelinated granule cell, which is also the slowest conducting.

Recurrent delay-paths expand the lengths of delays available to these systems. Recurrent circuits enable unbounded durations of neural delays for extensive, iterated processing. Multi-synaptic delay-paths that traverse multiple neural elements combinatorically expand the numbers of delays in a network. The brain is nothing if not a network of delay loops, and its recurrent, cyclic paths, properly configured, make unbounded, longer and longer, delays and processing sequences possible.

Spike coincidence mechanisms can be found widely in brain structures (cerebral cortex, cerebellum, and brainstems of many sensory pathways). Cortical pyramidal cells famously have numerous synaptic inputs, irregular spiking, and multivalent responses to diverse stimuli, which is consistent with the inference that relatively small subsets of well-timed incoming spikes are sufficient to cause these cells to fire (Softky and Koch, 1993). As a result, these cells may be better regarded as structured ensembles of millisecond-scale coincidence detectors, e.g., as with the Tempotron of Gutig and Sompolinsky (2006) or the multi-layered convolutional network of Beniaguev et al. (2021), rather than running integrators that average input firing rates over much longer timescales (Abeles, 1982). Complex dendritic coincidence trees would enable many different sets of synapses, each set having a specific connectivity and relative delay pattern, to trigger spikes in these cells, thereby allowing them to participate in multiple neural assemblies.

#### 3.2.3 Oscillatory networks

Other kinds of architectures are possible that could also potentially handle some types of temporal codes. Neural networks that consist of coupled neural oscillatory elements (Greene, 1962) or ensembles of many elements. Oscillator networks have mainly been studied in terms of their dynamical behavior rather than specific information processing functions. Neural oscillations on population-wide scales are generally not thought to convey specific information about attributes due to their variability and limited bandwidth, but may nevertheless facilitate many non-specific integrative functions by synchronizing neural populations through common and emergent oscillatory frequency modes (Cannon et al., 2014; Cariani and Baker, 2022). Such networks can support neural codes that depend on oscillatory phase offsets (Hopfield, 1995; Cariani, 2022; Cariani and Baker, 2022). By virtue of their oscillatory resonances, such networks can be used for analyzing incoming coarse temporal patterns (e.g., beat tracking for musical rhythms), inducing brain states (e.g., sleep stages) associated with particular oscillatory modes, producing temporally patterned outputs (e.g., rhythmogenesis in central pattern generators), storing memories in the frequency-domain (Longuet-Higgins et al., 1970), visual segmentation and binding (Baldi and Meir, 1990), or creating new neural periodicities through emergent oscillatory dynamical modes (see section “4 Time-domain waveforms, signals, and systems: common signal operations, holography, radio communications, and the brain”).

With incorporation of nonlinear, multiplicative and thresholding processes, oscillatory architectures can potentially handle complex pulse-coded time-domain signals and operations, though less directly than time-delay neural coincidence networks, where specific interspike intervals can be offset with corresponding delays. Some of these issues are taken up in section “4 Time-domain waveforms, signals, and systems: common signal operations, holography, radio communications, and the brain” and elsewhere (Cariani and Baker, 2022).

#### 3.2.4 Reimagining canonical neurocomputational structures

Architectures that include greater neuroanatomical, cellular, and biophysical detail have also been proposed that operate on complex spatiotemporal spiking patterns (as in the polychronous networks, spiking orders, and spiking order trajectories). For reverse-engineering, the problem of how neural signal processing works to realize informational functions, simplified neural models with limited parameters may be more useful. Although ever more neuroanatomical and biophysical details can be incorporated, basic functional principles can be obscured by complex, inscrutable dynamics.

Beyond general time-domain architectures, canonical neurocomputational structures such as cerebral cortex, basal ganglia, hippocampus, and cerebellum will need to be reconsidered as temporally coded correlation devices (cf., Marr, 1991). Traditionally these circuits were functionally conceived in terms of channel connectivities and activations, but these circuits can be reimagined in terms of interactions of spike timing patterns in synfire chains and cycles.

Cerebral cortex can be reimagined in terms of analysis, processing, and production of temporal patterns. Cortical-striatal-thalamic circuits have been traditionally regarded in terms of task-specific motor control, interval timing, and attentional gating of thalamic sensory and motor channels. More recently, the presence of short temporal latencies, precisions, and narrow time windows of striatal action have been recognized (Pouzzner, 2020). In addition to gating or facilitating relevant channels, attentional modulation can also plausibly be achieved by matching top-down target signals with incoming, bottom-up sensory data (e.g., matched filters through correlational amplification of specific incoming signals). Given high temporal precisions, striatal spike trains could provide temporally patterned top-down signals that selectively disinhibit (amplify) similar temporal spike patterns entering the thalamus from afferent sensory pathways.

The cerebellum has long been considered as a neural timing organ (Braitenberg, 1961, 2002; Braitenberg, 1967). In the cerebellum massive arrays of slow, unmyelinated parallel fibers the flat, dense dendritic trees of Purkinje cells strongly suggest functions as canonical temporal coincidence elements. Although the cerebellum was originally conceived mostly in terms of motor timing relations and real-time control of sensory surfaces, more recently more general timing and sequencing roles in perception and cognition have been proposed (Braitenberg et al., 1997; Rodolfo Llinas and Negrello, 2015).

The hippocampus has traditionally been regarded in terms of a recurrent, autoassociative rate-place architecture (Marr, 1971). However, as in the cerebellum, its arrays of recurrent fibers support systems of switchable delay-paths. These paths then enable time-delay operations capable of rebroadcasting specific temporal spike sequences that can function as temporal-coded memory traces. In this view hippocampal replay essentially broadcasts selected temporal memory traces to the rest of the brain. Time compression of these spike patterns also enables them to be used as anticipatory guides in real time for prospective action (see section “3.6.1 Temporal memory traces”).

### 3.3 Brains as purposive, goal-directed systems

Brains are purposive, goal-driven feedback neural control systems that have embedded evaluative mechanisms for assessing goal satisfaction and for weighing the urgency of competing goals. The term “goal” is used here in a broad sense to mean any state that a system is organized to preferentially seek. Animal nervous systems have evolved sets of internal, embedded goals that promote survival and continuation of the lineage. Most goal-related operations, as well as their associated planning, and steering functions are thought to be mediated by neural circuits in pre-frontal cerebral cortex (Miller and Cohen, 2001).

Basic, immediate, “primary” goals under neural control involve preservation of internal organismic integrity (homeostasis: oxygen, water, ionic, nutrient requirements, injury avoidance, and damage control through pain minimization) and avoidance of imminent external threats (predation and injury). Longer term, less urgent, “secondary” goals go beyond basic survival to include reproduction, rearing of offspring, exploration, learning, play, stress and uncertainty reduction, and socially mediated rewards. Learning processes entail tunings of neural assemblies that improve functions (more reliable goal attainment, better performance, and better predictions) and can progress in the absence of more salient primary goals.

Motivations (goals and drives), expectations, and rewards, as well as emotional and cognitive states, determine what prospective actions will be taken. Depending on the current external and internal contexts, such as perceived opportunities for goal-attaining actions and emotional state, any goal can take potentially take priority in driving behavior. Internal goals thereby focus attention, drive decision-making through signal competition, and trigger task-specific behaviors.

The temporal theory posits that the neural signal for each goal is a characteristic temporally coded pattern of spikes. The pattern is broadcast widely throughout the brain to activate task-relevant local subpopulations, circuits, and signals and to suppress those that are not. Characteristic goal and reward signals permit representations to include goals that co-occurred with objects, events and situations, thereby enabling memories to be accessed according to the goals they fulfilled. In producing and broadcasting a goal signal, the system also begins to access memory traces that contain that signal, and with them still other signals related to brain states and action sequences that occurred with its past attainment (Cariani, 2017).

The temporal theory is anticipatory and similar to predictive coding models (Friston, 2010; Seth, 2013; Seth and Friston, 2016; Friston, 2019) in that the storage and access of signals related to perceived objects, events, environmental situations, and internal goals and procedures forms an internal model of the perceived likelihood of the efficacy of some particular prospective action in the context of some particular situation.

### 3.4 Neural representations

Representations, as used here, are organized systems of neural codes that signify related attributes. See Lloyd (1989) for discussion of representations in neurally grounded theories of mind. Attributes are informational distinctions, neural activity “differences that make a difference.” Distinctions constitute alternative functional states of the system that lead to different subsequent internal states and/or behavioral outcomes. Representations can involve attributes that vary along one dimension (e.g., loudness, lightness, and the pitches of notes on a piano), a few dimensions (e.g., timbre, color, texture, and odor), or many dimensions (the neural description of a physical object that includes basic sensory attributes as well as conceptual classifications, word labels, perceived affordances, emotional valences, and memories of similar objects).

Representations of objects, events, situations, and procedures are groupings of neural signals into unified, chunked, entities. We will refer to these represented entities as “composites.” Objects are collections of neural signals associated with sustained co-occurring attributes. Objects can include collections of perceived external attributes of particular physical objects (e.g., a specific fork) or collections of constructed internal attributes that constitute concepts (e.g., the concept of a fork). Objects are atemporal in that they are not defined primarily in terms of discontinuities that are marked in time – they do not have beginnings, ends, and durations. In contrast, events are collections of attributes that are demarcated in terms of neural time markers. Events have beginnings (onsets), ends (offsets), durations, and specific timings. Events can also contain mixtures of external and internal attributes such as a knock on the door, the emergence of a thought, or the recall of a memory. Situations are collections of attributes related to contexts, again either external or internal. These can be ongoing and quasi-stationary, as with objects, or emergent and episodic, as with events. Procedures are internal attributes associated with neural signals and signal sequences, such as in motor programs and trains of thought.

The time domain theory proposes that virtually all of these attribute distinctions are encoded in characteristic temporal patterns of spikes, be they within spike trains of individual neurons, spike volley patterns produced by neural ensembles, or spike timing correlations in populations.

#### 3.4.1 Vectorial representations of multiple attributes

In this theory, the dimensional structure of mental representations mirrors the correlation structure of the neural codes. For sensory representations this structure comes directly the stimulus in phase-locked sensory systems or from characteristic stimulus-triggered spiking patterns in non-phase-locked systems. Due to their iconic nature, the various temporal patterns produced by phase-locked systems can usually be related by some continuous deformation (affine transformation) that provides spaces of correlation-based perceptual similarities and differences. For example, the distributions of interspike intervals that encode nearby musical pitches (musical C_3_ vs. D_3_) overlap with each other (Cariani, 2019, Fig. 5.8). On the other hand, the divergent correlation structures produced by different sensory modalities, such as visual forms or smells, result in discrete, separate dimensions that highly independent of each other.

Different, independent attributes are encoded using different, orthogonal temporal patterns such that combinations of attributes can be represented as vectors. Each dimension encodes one attribute which has a characteristic set of temporal pulse patterns that indicate its signal type. For example, a musical note has a set of distinct, highly independent attributes that include loudness, duration, apparent location, F0-pitch, pitch height, and several dimensions of timbre. The theory posits that each one of these attributes is represented by characteristic neural temporal patterns of spikes that indicate which attribute is being distinguished (e.g., F0-pitch vs. loudness) and the specific value of that attribute (e.g., middle C).

The temporal coding thus permits the form of the neural signal to indicate its signal type, i.e., which attribute the neural signal signifies to the rest of the system. Because the signal type and specific value are no longer bound to particular neural channels or transmission paths, as they are in channel-domain networks, neural signals can therefore be liberated from particular nodes and wires. This in turn enables broadcast of signals and selective reception by distant neural assemblies that are tuned to respond to different signal types.

In the temporal theory, the strength (salience) of the neural signal associated with each attribute at any given time is indexed by the relative prevalence (fraction) of the characteristic temporal pattern associated with each attribute currently being produced within a given neural population or larger network. It can be described in terms of a positive scalar (ranging from 0 to 1) reflecting the fraction itself, or binary (0 or 1) reflecting whether its fraction exceeds some threshold of significance.

At any given time, the current functional, representational state of a local neural population or global network is the set of neural signals being produced (regenerated within) that system. This can be described in terms of *N* attributes (types), *M*_*i*_ alternative distinctions (values) for each attribute *i*, and the strength of each attribute-value combination. The resulting vector has *N* × Σ *M*_*i*_ dimensions with one signal strength-salience-prevalence-intensity scalar for each dimension. As there are typically large numbers (thousands) of discriminable attribute values for each external attribute, for cognitive internal attributes, and mnemonic distinctions, as well as those for emotional and motivational states, the total number of possible alternative functional states of the system is quite large.

This signal-based vectorial representational system can support analogies, i.e., that disparate entities are similar in some ways (common dimensions in signal space), generalizations, i.e., members of sets of entities that share several common attributes (multiple signal dimensions in common) and interpretability, i.e., how they are similar (what attributes the common signals connote).

Because this set is a small subset of possible neural signals, both in terms of numbers of dimensions (attribute signal types and of distinctions within each dimension (attribute values), the representational state vectors are very high dimensional (reflecting all possible attributes) and sparse (with only a small number of attributes and their associated spike train signals in play at any time). These kinds of high-dimension vectorial representations have many advantages for implementing concept dynamics, search/retrieval processes, and analogical reasoning (Widdows, 2004).

#### 3.4.2 Binding: organization of attributes into separate, unified wholes

Beyond simple vectorial collections of attributes and signals currently in play, there is further organization that binds subsets of attributes into unified, separate, largely independent, objects, events, situations, and procedures.

The organization of perception has traditionally come under the rubric of Gestalt processes, segmentation and binding [the “binding problem” (von der Malsburg, 1981/1994; von der Malsburg, 1995b)], and scene analysis. The clearest, most obvious examples are found in vision and audition, with many parallel principles and analogous transformations between the two modalities. In vision, regions of images with similar forms, textures, colors, and correlated timings and motions group together. In audition frequency components group by common onsets (roughly synchronous event timings) and common subharmonics (harmonic relations). Hearing out separate streams of related events in music (polyrhythms and polyphony) and speech (“the cocktail party problem,”) involve separation of independent streams (voice lines, instruments, and speakers) and grouping together of events related to one or another stream. Within each stream are also groupings and separations of events in time (“chunking”). Events close in time tend to group together (the Gestaltist Proximity Principle): notes into musical phrases and phonetic sequences into syllables and words.

Similar binding processes also exist in cognition to group features into categories (concepts), in memory to group related events into discrete episodes, and in the orchestration of action to group specific movements and action sequences into wholes.

The temporal theory posits that bindings are realized through signal interactions. Signals with correlated timings and/or common subpatterns of pulses interact in delay-coincidence networks to reinforce each other (Figure 1B; Cariani, 2001a) and to create new temporal patterns that are related to their co-occurrence (see section below).

This theory is similar in many respects to Malsburg’s general theory of correlation-based perceptual and cognitive organization (von der Malsburg, 1981/1994; von der Malsburg, 1995a), but more explicitly asserts that the correlation relations are temporal correlations amongst spikes. There are also similarities to models of binding based on temporal synchrony (Singer, 1999; Engel and Singer, 2001). Whereas Singer’s mechanism for binding is based on temporal synchronies of channel activations, the time domain mechanism is based on temporal pattern similarities that do not necessarily require precise (zero-lag) synchronies: in the temporal pattern theory temporal proximity is sufficient for binding. As long as the similar patterns arrive at a given location within temporal integration windows of tens to hundreds of milliseconds, they can still be bound together. Because near simultaneous events also create characteristic patterns of signal interaction temporal pattern representations can also incorporate response synchronies. The two kinds of binding principles are therefore not at odds with each other.

#### 3.4.3 Formation of new signals that signify bound entities

Grouping requires some process that binds the various attributes that constitute a unified whole that can be represented and handled as a separate entity. The whole can be an object, event, situation, or procedure. In the time domain theory, binding is accomplished through signal interactions that produce new signal patterns that are characteristic of the whole, such that patterns that emerge from the relational interactions of the parts are different from simple superpositions (additions) of the constituent signals. The whole is more than a simple combination of parts.

The new, emergent signal patterns then can become tags that signify the whole entity (Cariani, 1997, 2012). They then function as markers for compositions (bindings), variously conceived in terms of concepts, cognitive nodes (MacKay, 1987), perceptual symbols (Barsalou, 1999), semantic pointers (Eliasmith, 2013), and meta-representations (Lloyd, 1989). Our perspective is in general accord with grounded cognition (Barsalou, 2008; Avarguès-Weber and Giurfa, 2015): the same phylogenetically ancient binding and composition mechanisms subserve perception, cognition, executive functions, memory, and the organization of action.

Activation of a cognitive node involves activation of a specific neural assembly that produces a characteristic composite signal. The composite signal contains both temporal patterns related to the constituent features of the cognitive node and the new, emergent pattern that connotes their composition, i.e., the whole node. This allows for bottom-up activation of higher level nodes and production of their tags by virtue of the co-occurrence of subsets of signals related to their constituent features.

For top-down access to the constituent signals and their associated attributes, a process by which the emergent composite tag is formed needs to be at least partially reversible. By injecting signals related to node tags, nodal neural assemblies in turn can produce the signals that signify the constituent features, i.e., top-down activation of basic features. It enables comparisons between categories, e.g., how a cat is like a dog. By activating the two nodes, their constituent signals are all produced and the signals related to attributes the two cognitive nodes have in common mutually amplify each other.

The new composite signal then interacts with all other signals in current circulation, activating other nodes that share temporal pattern components in a process of spreading activation (Collins and Loftus, 1975; Anderson and Hinton, 1981). The process is highly parallel, with all signals in local brain regions and in global circuits interacting at once. Eventually a stable set of mutually reinforcing signals emerges from the signal dynamics of reinforcement and competition. The same grouping and compositional processes that form representations of unified objects, events, situations and procedures may subserve higher level cognitive concept dynamics, executive planning functions, and motoric orchestrations.

If through repeated exposure, rewarded or unrewarded, new neural assemblies become configured to respond to the tags, then in essence new signal dimensions, i.e., new primitives (Cariani, 1997, 2012) have been added to the system. The tags become markers for new attributes that distinguish between composites that have particular signal combinations from those that do not. They enable classifications based on common feature combinations, which is essentially another form of grouping by common time structure.

Several possible means of producing new signal patterns in an open-ended way are discussed in section “4 Time-domain waveforms, signals, and systems: common signal operations, holography, radio communications, and the brain.” Nonlinear processes, such as signal multiplication (mixing) and thresholding (rectifications and triggers) operations produce new characteristic patterns. This is most simply seen in the frequency domain, where mixing operations, e.g., modulated carriers of radio, produces new frequencies that were not present in the constituent signals (see section “4 Time-domain waveforms, signals, and systems: common signal operations, holography, radio communications, and the brain”). In the time-domain, multiplication of pulse patterns that constitute different signals similarly can produce new patterns.

If the operations afforded by nonlinear signal interactions permit inversion, i.e., extraction of constituent signals from their interactive mixtures, then both bottom-up formation of perceptual symbols and the top-down extraction of constituent signals (features) and their attributes can be realized. Convolution is one operation that enables these partially reversible bottom-up top-down operations (Longuet-Higgins, 1989), but its time domain implementation may require some signals to be reversed in time (as the existence of reverse hippocampal replay might suggest). Alternately, similar operations might be carried out using temporal cross-correlation operations involving simpler addition, subtraction, multiplication, and thresholding operations (see section “3.4.3 Formation of new signals that signify bound entities”) that are quite straightforward for delay-coincidence networks.

### 3.5 Signal dynamics: mutual reinforcement, competition, and spreading activation

In the time-domain theory the functionalities of Table 1 and the operations of Figure 1 are carried out by interactions of temporally patterned signals (signal dynamics). The theory parallels many conventional neurocomputational theories except that the functional states of the system are time domain signal productions rather than patterns of neural channel activations.

Cognitive nodes, in the time-domain theory, are implemented by signal productions. Following some triggering event, such as an external stimulus, neural signals are produced that have temporal structure that encodes particular features (attributes). The signals course through pathways, interacting and sequentially activating neural assemblies that, through training, are sensitive to their temporal structure. In turn these assemblies emit similarly patterned signals that reinforce the incoming ones. They may also emit characteristic tag signals of their own (Figure 1E) that signify the co-occurrence of specific sets of features. These signals propagate to activate yet other assemblies in a “spreading activation process” (Collins and Loftus, 1975; Anderson and Hinton, 1981; MacKay, 1987). Here cognitive nodes are sets of signal productions by neural assemblies (sets of interacting neurons that operate together to realize some function). Multiple neurons can form neural assemblies, and each neuron can participate in multiple assembles.

### 3.6 Memory

Memory is integral to virtually all behavioral functions. Neural memory mechanisms maintain, store, access, and retrieve records of past internal neural events related to all aspects of experience (perceptual attributes of objects and events, cognitive states, affective and motivational states, event sequences and timings, and rewards). The memory traces encoding these various aspects can then function as internal models of event correlations that can positively guide prospective action. The purpose of remembering the past is to anticipate the future so as to more wisely choose actions that have been found successful in similar past situations.

#### 3.6.1 Temporal memory traces

The temporal theory proposes that information is stored in the form of temporal memory traces. These are held to be temporal patterns of spikes associated with all attributes of objects, events, and situations, including their relative sequencing and timing as well as internal orchestrations of action, and any rewards or punishments that ensued (Cariani and Baker, 2022; Cariani, 2017). Memory traces consist of the same multiplexed, multitemporal patterns that originally encoded this information. Temporal coding lends itself to direct representations of event timings because of widespread spiking and bursting responses at event onsets and offsets. We have proposed a general temporal coding framework by which all event attributes might be encoded by characteristic spike latency patterns (Figure 1A; Cariani and Baker, 2022).

Such a framework enables tape recorder-like memory mechanisms for both storage and readout of temporal memory traces that contain event timings, sequencings, and specific attributes. If the tape recorder mechanism can be sped up, then faster-than-real-time readouts of internal events and their ultimate consequences can be accessed. There is a sizable literature on hippocampal replay processes (Foster, 2017), which resemble such tape recorder memory mechanisms. Replays both forward and reverse in time have been found in both sleep and waking states of rodents, monkeys, and humans. Studied mainly through rat maze running experiments and recording of place cells, their contents correlate with recent experience, salient but less common experiences in the recent past, or even alternative paths that were never taken. Replay appears to be not only about the replication of past event sequences. Various theories posit their function in terms of memory consolidation, reinforcement learning, and cognitive maps.

An advantage of temporal memory traces is that the timing and sequencing of events is an integral part of the representations that are stored. Not only are the events recorded, but also when rewarding and reward predicting events will be expected to occur. This comports with the behavior of dopamine neurons in reward timing prediction (Balsam et al., 2024). Behavioral evidence from conditioning experiments suggests that humans and animals readily construct timelines of multiple events that occur at predictable timings relative to each other (Miller and Barnet, 1993). The timelines of whole event sequences can be constructed even when only pairs of events are presented. This not only points to a general mechanism for assembly of memory traces from fragments but also the assembly of a unified timeline from the relative timings of the individual events. For a mechanistic theory of the construction of timelines based on “time cells” using Laplace transforms (see Howard et al., 2015).

Whereas psychological treatments of memory (e.g., Howes, 2007) focus mainly on the kinds of information that is stored (e.g., declarative-semantic, episodic-autobiographical, procedural, categorical recognition, verbal, echoic, iconic-visual, haptic, and emotional memory), we focus here on their general neural substrates. Modality-specific contents of memory traces appear to reside in modality-specific brain regions, where their attributes are represented in terms of modality-specific local codes. Supramodal contents, such as event timings, are held to be distributed over much wider sets of brain regions and to be represented in terms of global codes. This means that the complete memory trace of an event, object or situation with multimodal attributes is not available at any one location, and instead is distributed across brain regions.

The spatially distributed nature of the information in turn necessitates a process of either assembling the various local fragments into a whole or binding together the local and global parts such that all aspects of the trace can be accessed and activated. This process has been termed “hippocampal pattern completion” (Horner et al., 2015). In the time domain theory, because the supramodal temporal patterns are available everywhere, their common temporal patterns co-occur with locally coded patterns, which provides a basis for binding based on temporal correlation. This pattern completion process takes time, as the fragments are activated, their signals begin to be produced, and related patterns are then coactivate. This is an iterative assembly process may explain why some memories are assembled and retrieved quickly, whereas others, particularly episodic sequences of events and their details, may take much longer. In general, the more frequent a stimulus is presented to the system, the more recent the presentation, the more valued any associated reward, and/or the more perceptually salient (e.g., surprising and unpredicted) it is, the more neural assemblies will be tuned in some way to resonate with it. The more neural assemblies participating in producing fragments of a full memory trace, the shorter the latency of neural responses, and consequently the faster the signals produced by these assemblies will enter the signal space to interact (amplify or compete) with incoming and already circulating patterns.

As in dual-store theories of memory, the time-domain theory assumes that there are two types of neural processes that store, maintain, and retrieve temporal memory traces. These are a short-term, temporary reverberatory memory (STM) and a long-term (quasi-) permanent memory (LTM). In hippocampal replay, the hippocampus broadcasts rewarded and salient contents of STM to the rest of the system, during both sleep and waking states, where repeated presentation causes synaptic and possibly other changes that create permanent LTM records.

Both short-and long-term memory stores are content-accessible in that temporally coded incoming signals activate and amplify circulating memory traces with similar temporal structure, and vice versa. Memory is thus regarded as a temporal pattern-resonance process. If circulating signals and memory traces both share temporal structure, then there is no need for “pointers” or physical addresses of items in memory. The form of the message is its own pointer that permits the contents of a memory trace to be accessed (addressed).

#### 3.6.2 Short term memory

Short-term memory (STM) actively regenerates incoming spike patterns that constitute temporal memory traces in reverberatory delay paths. Short-term reverberatory memory is a temporary store that functions as an erasable buffer for recent sensory inputs (echoic, visual-iconic, and haptic memory) and internal functional states (working memory). It is temporary (labile) in that it does not persist if interrupted by anesthesia, coma, sleep, or subsequent strong, overriding signals (distractions and masking inputs).

In the time-domain theory, the neural mechanism supporting short-term memory is active regeneration of circulating neural signals in facilitated reverberatory delay loops. The mechanism enables rapid response because the circulating neural signals conveying memory traces can interact directly with incoming signals. The circulating signals provide a recent context for the incoming signals to be processed.

By themselves, the network delay paths that constitute the loops do not favor any particular incoming signal over any other. However, if particular trace signals are already circulating in reverberatory loops, then they will interact with incoming signals with similar temporal structure to build up circulating memory traces. Differences between circulating and incoming signals can also generate new mismatch signals that enter delay loops. The phenomenon of mismatch negativity (MMN) is highly suggestive of this kind of running, comparative, temporal correlational process. Given the astronomical number of alternate delay paths, any incoming temporal pattern of spikes can be propagated though the delay networks.

Regeneration of signals in these loops can be dynamically enhanced by bursting activity, transient activations of NMDA receptors, and ensuing STDP processes that modulate synaptic efficacies based on recent spike correlation history (Markram et al., 2012). Signals in the loops can be attenuated through inhibitory inputs. How long a neural signal persists in delay loops (delay paths through global networks and local circuits) depends on loop gains. In most circumstances, loop gains are slightly negative, resulting in gradual attenuations of signals in reverberatory delay loops. However, when the loop-gains are positive, signals build up in neural circuits and spread across networks. Loop gains can be moved from negative to positive when incoming signals are strong and self-reinforcing (periodic) or when neural populations that participate in the loops are disinhibited (attentional mechanisms).

#### 3.6.3 Long-term memory

Long-term memory (LTM) is a nonlabile, permanent store that can, in some cases, persist over the lifespan of an individual. The temporal theory presumes that this type of memory is supported through neural assemblies trained to respond to and/or to produce specific sets of spike temporal patterns. Hippocampal replay of neural correlates of temporal sequences of events in activations of place and time cells is widely appreciated (Eichenbaum, 2016). Less well known is the phenomenon of the “assimilation of rhythm” that is observed in single units, whereby a neuron repeatedly electrically stimulated at a particular pulse frequency will reproduce the rhythmic pulse pattern when subsequently activated (John, 1967; Morrell, 1967; Bogdanov and Galashina, 2012) Either individual neurons themselves and/or neural assemblies to which they are connected have the capacity to produce temporal patternings that are repeatedly presented to them.

Acquisition of such long-term temporal pattern resonances could be mediated by two types of mechanisms. As in current mainstream thinking, long-lasting changes in synaptic efficacies (“weights”) associated with specific sets of interneural delays, as in TDNNs, can tune neural assemblies. Another possible type of mechanism encodes neural activity patterns in molecular modifications of microtubules or of stable polymers. Molecular mechanisms that could potentially provide a durable physical basis for encoding engrams were proposed in the 1960s (John, 1962; Landauer, 1964; John, 1967; Byrne, 1970), but following controversies over related planaria-based experiments, the field was defunded and rendered dormant for several decades. More recently, these hypotheses have been reconsidered and refined (Landry et al., 2013; Gallistel, 2017; Langille and Gallistel, 2020; Gallistel, 2021; Gershman et al., 2021). Molecular mechanisms for encoding time sequences in spatial patterns of sidechain markers along the lengths of nucleotide polymers are also conceivable (Cariani, 2017).

In the temporal theory, temporally compressed, faster-than-real-time temporal patterns are widely broadcast to subsequently become fixed into temporally compressed memory traces. The sped-up patterns can serve as predictors of the future. Because the traces hold representations of early events in some remembered sequence of salient events, the traces can be activated when situations similar to past ones arise. Because the traces hold the sequences of encoded internal processes, they can serve as triggers for replicating action sequences. Because they also contain neural reward signals related to the consequences of prospective actions, they can serve as guides for whether the same action sequences should be facilitated and replicated (positive reward) or inhibited and avoided (negative reward) this time around. Because memories link action sequences with goals (rewards) and these are both stored in content-accessible form, injection of signals characteristic of particular rewards can call up related actions, and vice versa. This may be the basis of how mirror neurons behave. Observing another animal’s actions activates one’s own memory traces that contain similar actions that in turn through pattern completions retrieve whole action sequences and the rewards that were obtained.

Reverse replay sequences encode reward states first, so this permits searching for what prospective actions might lead to a particular reward by sending that reward signal and activating internal and external action sequences that led to it in the past. This kind of mechanism is simple, not requiring elaborate internal models, and universal. Even in the absence of significant reward or goal-directed action, such a mechanism can also record correlations between environmental events (external attributes) to extract causal linkages.

The memory is associative, storing individual items and also their combinations. Both memory stores are content- or pattern-addressable (content-accessible) in that the memory traces that are circulating and/or produced interact directly with other incoming neural signals. If an incoming signal has some similarity with that of a memory trace (common subpatterns), then both the trace and the incoming signal are amplified (mutually reinforced). This enables content-based search processes. By injecting a signal related to some attribute of interest, all of the memory traces that contain temporal patterns associated with that attribute will eventually be activated. A series of spreading activations ensues as other related nodes are activated in turn. Those neural assemblies that are highly tuned to specific patterns due to repeated, frequent and rewarded presentations will respond earliest (fast response memory). Examples would be the phonetics and words or printed characters of one’s native language. Those memory traces that are seldom accessed will have weaker responses such that it may require an iterated slower process for them to be activated.

## 4 Time-domain waveforms, signals, and systems: common signal operations, holography, radio communications, and the brain

Waves are fundamental. Their mechanical and electromagnetic forms and interactions dynamically define our universe and our perceptions of it, from the Big Bang, to seeing and recognizing each other. Starting from basic principles, we take a time domain perspective to describe how simple physical laws applied to waves, may explain many of the functional mechanisms of brain observations and behaviors. We focus on neural spikes, codes, dynamic interactions and integrations, communications, etc. operating in the context of self-organized, recurrent, massively distributed, parallel processing network architectures. Neural networks are effectively, hybrid analog-digital systems characterized by a combination of linear and non-linear operations. The signals by which they respond to complex stimuli, process and communicate information to achieve their functions [see section “2 Basic brain functionalities (what is to be explained),” Table 1], i.e., perception, motor control, cognition, decision making, etc., are simply spikes and (not so simply) spike patterns.

### 4.1 Temporal perspective leads to emergent processes

We take a temporal perspective to understand and explain primary brain functions and mechanisms. The system uses patterns based on precise spike timings, e.g., interspike intervals. These are integrated and coupled through phase-sensitive wave dynamics, to drive brain mechanisms for perception, motor activity, memory, as well as cognition, executive functions, etc. We hypothesize that plausible brain mechanisms may be employing holographic- and radio-like principles, with neural systems performing common mathematical operations (auto- and cross correlational, convolutional) inherent in these. These principles may help explain the integration and coordination of multimodal information flow across the cortex, and predict the natural emergence, elimination, and sequencing of routinely observed oscillations and other neural phenomena.

We suggest that holographic and radio communication principles, employing correlations (auto/cross) may be applicable much more holistically, not only to memory models and pattern recognition specifically, but much more broadly to sensory, memory, cognitive, decision making, and affective processes, in general.

Adopting a temporal perspective, *per se*, is not new. It has an extensive and distinguished history (summarized below) which identifies and associates how wave dynamics support common holographic and radio communication principles. What is new is the extent to which we hypothesize and propose that temporal principles and laws of physics, may determine, consistently and coherently, many brain functions and behaviors.

Much of the underlying science for the basic understanding of the principles supporting our temporal perspective, builds on notable research findings, both theoretical and experimental, from 50 or more years ago. We contend that many empirical observations, of neural behaviors both locally and globally, may rather simply, emerge from these basic principles!

We briefly review this work to show how it motivates this time-domain signal-centric theory. We are “standing on the shoulders of giants” and their many decades of pioneering research to support this vision. The work of Lashley (1958) demonstrated broadly distributed, non-localized neural processes and memory traces (Eichenbaum, 2016), “mass action” and modifiable “equipotentiality.” See reviews Orbach (1998), Nadel and Maurer (2020). Extending it, Hebb (1949) proposed principles of neural assemblies (Eichenbaum, 2018), plasticity (Markram et al., 2012), synchronized neural ensembles, and phase sequences, notably asserting the general organizing principle that neurons that fire together, wire together (Nadel and Maurer, 2020; Almeida-Filho et al., 2014).

What follows is a discussion of these principles, including a timeline of some relevant milestones and influences, primarily starting in the 1940s. These contributions span multiple disciplines, especially drawing on neurophysiology, physics (biophysics), experimental and mathematical psychology (psychophysics), and mathematical modeling. Experimental and theoretical threads are interwoven. Some of these pioneers worked on experimental research, some exclusively on theory, and others on both. Many were trying to figure out how brains and neural systems work. Others were focused on other disciplines, especially signal processing, holography, radio communications, etc. The temporal perspective presented here draws on all of these, and more.

#### 4.1.1 Common correlation and convolution operations

It starts with how information contained in waveforms is represented by signals and how these are processed in the time and/or frequency domains. The two domains are formally equivalent, such that any signal representation or operation in one has an exact counterpart in the other, but their neural implementations may be very different. The information in a digitized waveform is identical, but decomposed differently in the two domains. In terms of neural signals, the time-domain decomposition consists of individual spike responses, whereas the frequency domain decomposition describes neural responses in terms of sinusoidal Fourier components.

As is evident in following sections, the mathematics relating wave dynamics, to pattern coding, pattern detection, retrieval, reconstruction, and holographic principles, share common numerical operations, especially correlation and convolution operations. These operations are tightly related between the frequency and time domains: multiplication in frequency corresponds with convolution in time (Smith, 2003). Because computation of a Discrete Fourier Transform (DFT) and multiplication of its complex spectrum is much more computationally efficient than a time-domain convolution, correlations made in the time domain are typically transformed to the frequency domain using the Fast Fourier Transform (FFT), the spectra are multiplied, and then transformed back to the time domain using the Inverse Fourier Transform (IFT).

Although Fourier analysis often offers substantial computational convenience, it is seriously disadvantaged in that it imposes a window of time over which both high resolution time and high-resolution frequency information are not simultaneously available (uncertainty principle). This windowing requirement inherently limits its resolution in characterizing aperiodic and rapidly changing waveforms.

The order in which correlational operations are performed is important for maintaining precision in sequential relationships. Despite the similarity otherwise in convolutional computations, a reversal or flip of temporally ordered parameters is required for convolutions. Therefore, convolutional operations are commutative; correlational operations are not. In the time-domain, we largely focus on auto correlations and cross correlations of signals and sequential patterns of these that maintain temporal ordering.

Early in his prolific career, Licklider and Pollack (1948) and Miller and Licklider (1950) conducted a series of psychoacoustic experiments, demonstrating the high intelligibility of infinitely peak-clipped speech. This demonstrated a proof-of-concept for the preservation of speech information, using a transformation similar to neural phase-locking. His work on speech masking (Licklider, 1948) underscored the critical role for precision phase-locked spike timings. His auditory models combined cochlear filtering with correlation operations that used phase-locked spikes, tapped delay lines and coincidence detectors. The earlier duplex model (Licklider, 1951) implemented an autocorrelation analysis for pitch, whereas the later triplex model added cross-correlation operations to account for binaural perception (Licklider, 1959) as well as a self-organizing central processor.

Licklider (1951) observed that the “basic operations of autocorrelational analysis are delay, multiplication, and integration. The nervous system is nicely set up to perform these operations. A chain of neurons makes an excellent delay line. The spatial aspect of synaptic summation provides something very close to multiplication. And the temporal synaptic summation is essentially running integration.” These observations still ring true today.

In the same timeframe, Meyer-Eppler extensively explored applications of auto- and cross-correlations to characterize signal processing for speech, music, radio communications, and information theory (Meyer-Eppler, 1953; Lange, 1967).

#### 4.1.2 Correlational models

In addressing the need for understanding global mechanisms by which brain cells can be selectively activated and deactivated, von der Malsburg (1981/1994) proposed a “Correlation Theory of Brain Function.” He stressed that correlations of neural activation patterns for fine temporal structure (e.g., spike trains and spike bursts) could characterize and drive the structure and coordination of brain dynamics. Parallel temporal correlations could form the basis for a series of sequential synaptic modulations, neural switches, channel selectivity, and neural plasticity for short- and long-term memory mechanisms. Such mechanistic processes are efficient and enable high-capacity memory storage. In discussing single neuron limitations with respect to firing rates, noise variability, etc., he proposed that “composite elements” (neural ensembles) could overcome these to establish correlation driven “specialized connectivity patterns” and support “global brain organization.” Although not offering an explicit algorithm, he explained how interacting spikes may act as coincidence detectors, and enable synchronous neural binding in an approximate range of 1–10 msec (von der Malsburg, 1999).

Autocorrelation and cross-correlation-based mechanistic arguments are well-founded. A time-domain brain theory relies on the importance of high temporal resolution mechanisms for global brain organization and function. Poorer temporal resolution measurements and analyses necessarily afford poorer approximations.

#### 4.1.3 Coding waveforms to preserve high resolution time, amplitude, and phase information

As described in section “3.1,” temporal coding can take different forms. Temporally coding and processing of neural interspike intervals, directly captures detailed spike train information. Spike trains and bursts expressed as temporal interspike intervals and spike order codes, can capture unique patterns, and can be represented as time series and manipulated with vectors and matrices (Gautrais and Thorpe, 1998; Xie et al., 2024; see section “3.4.1 Vectorial representations of multiple attributes”).

In the 1960s, the neurophysiologist Jerome Lettvin designed a simple electronic device to generate and record logarithms of interspike interval durations (Chung et al., 1974). When a fixed frequency sinusoid was combined with thresholded band-limited noise, “a pattern reminiscent of interference fringes emerges…such bands of preferred and forbidden interspike intervals are frequently encountered in endogenous activities of neurons in the central nervous system. Rhythmic bursts of impulses reveal the interburst interval as well as the range of interspike intervals within each burst.” Lettvin (1970) referred to this analysis in terms of radio signal processing, as a kind of “single sideband sampling theorem” (see section “4.3 Radio communications principles generate emergent brain signals”).

This time- or phase-locking analysis is a unified representation that can be applied to arbitrary mixed signal waveforms, irrespective of their periodic or aperiodic nature. Phase-locking analysis applies to any kind of waveform (e.g., acoustic, electromagnetic, seismic, etc.), as well as to neural spiking patterns, both for fine millisecond timescales and for population-wide responses on much coarser timescales. Because such analysis characterizes neural responses, as well as speech, music, and other waveform signals, it can easily be used to relate these to one another. Time intervals are computed phase-consistently (e.g., peak amplitude and zero-crossings) between successive waveform cycles. Excellent temporal, amplitude, and phase resolution is preserved. This is especially useful for precisely detecting signal discontinuities (e.g., onsets and offsets) and for characterizing transient events of short duration (millisecond and sub-millisecond) (Baker et al., 1972; Baker, 1975, 1979). Such discontinuities frequently act as segmentation markers (state of change indicators) between successive signal states.

As discussed in section “3.1,” neural phase-locking is a broadly observed synchronous response to both external stimuli (acoustic, visual, tactile, etc.) as well as to internal events (respiratory, cardiac, etc.). Many sensory and sensory-motor systems phase-lock to diverse stimuli to produce correlated spike patterns. These patterns can be easily induced and assimilated to generate corresponding replication/synchronization of motor actions (e.g., finger/toe tapping and dancing). Furthermore, such time-domain patterns are readily observed and conserved across a number of animal species (e.g., parrots, Asian elephants, apes, and sea lions). The high temporal, amplitude, and phase resolution of phase-locked signals enable their correlational relationships and interactions, to be precisely measured and assessed. As such, it can be a powerful tool for analyzing many stimuli as well as spike trains and spike bursts, for synchronization, coherence, oscillatory dynamics, and other characteristics.

#### 4.1.4 Phase matters: interference and delay lines

There are many ways of modifying information at synapses, with excitatory and inhibitory neurons interacting, neurotransmitter effects, etc. Another simple mechanism directly arising from wave dynamics, is to combine two waveforms in different phase relationships to one another. For example, consider simply summing two waveforms of the same frequency, either totally in phase to gain max amplification (constructive interference), or combining them in antiphase (destructive interference), where the waveforms completely cancel each other out, thereby fully suppressing both. Combining these two waveforms at different relative phase relationships will render intermediate results.

The *same* signal transmitted (broadcast) over two or more pathways with different delays (e.g., via direct and/or indirect pathways) can be combined together at a later stage such that relative to any of the interacting waveforms, the resulting shifted waveform will accordingly be amplified, suppressed, or partially modified. The consequences of such wave dynamics could potentially have major effects on our understanding, and ability to control (e.g., inhibit and amplify), multiple neural functions and behaviors. For example, such a delay line mechanism could plausibly operate to enhance or suppress selective attention. Research to assess how such mechanisms work, can best be performed when raw waveform data is available.

Phase alignment and coordination, via constructive and destructive wave interference dynamics is a standard control mechanism for establishing the likelihood of combining or suppressing signal amplitudes in communications signal integration, and signal propagation (e.g., traditional echo cancellation technique for long distance landline telephone communications).

Positive phases are excitable (“windows of excitation”); negative phases are suppressive (“windows of suppression”). The relative phase relationships, or degree of coherence, between interacting signals can linearly control the degree of interaction.

Furthermore, the phase relationships themselves can also be directly modified by the presence, absence, or shifts of time-delays in the interacting signals (e.g., combination of multiple direct and or indirect signals). Delay lines produce phase shifts. Interactions are further controlled by a variety of nonlinear processes; threshold mechanisms (e.g., “integrate and fire”), neural competition (e.g., “winner-takes-all”), neural mixing (see section “4.5 Radio cascade neural mixing model”), system resets, etc.

#### 4.1.5 Phase matters: effects on neural signals

Many lines of research have explored the evidence and effects of phase interactions. Hirsh (1948) discovered that phase differences were the critical factor in discriminating signal from noise with binaural presentations of signal plus noise in one ear, contrasting with just noise in or out of phase, in the other ear. Applying cross-correlations, Jeffress et al. (1956) demonstrated how to detect and localize acoustic signals both bi- and monaurally.

Working with macaques, Canolty et al. (2010) demonstrated oscillatory phase coupling in single neurons and neural ensembles, across multiple brain regions, both distal and proximal. Using transcranial stimulation, Dugue et al. (2011) asserted that oscillatory phase mediated human visual perception. McAfee et al. (2019), simultaneously recording in cerebellar Purkinje cells (mouse), medial prefrontal cortex, and hippocampus, found differential frequency-sensitive phase relations in coherent oscillatory interactions between these, indicative of temporal coordination.

Ten Oever et al. (2020), found evidence that different oscillation phase differences modulate human discriminations of different pitch stimuli. Using ferrets, Galindo-Leon et al. (2024) reported causal phase- and amplitude-coupled interactions, by analyzing similarity measures across multiple ECoG channels, tested across different cortical brain regions. Their human resting-state MEG studies reproduced these results, as did computer model simulations. There is also now evidence that diverse anesthetics causing loss of consciousness, disrupt the phase alignment in cortical oscillations (Bardon et al., 2024).

In a small study of subjects suffering from sleep onset insomnia, the administration of acoustic pulse stimuli delivered antiphasically to subjects’ alpha oscillations resulted in a reduction in the time it took for them to fall asleep (Bressler et al., 2024).

#### 4.1.6 Phase matters: synchronization and selective attention

Phase relations, phase-shifts, and time-delays determine the degree of phase synchronization of interacting signals, both constructively and destructively, to variously facilitate or inhibit active pathways. Evidence of these phase interaction and synchronization effects have been broadly observed across the brain, in sensory, motor, cognitive, and behavioral regions (Singer, 1994; Sauseng and Klimesch, 2008; Kopell et al., 2010; Kopell et al., 2011; Schmidt et al., 2013; Stetson and Andersen, 2014; Fries, 2015; Sacchet et al., 2015; Fischer, 2021; Gupta and Bahmer, 2021). Specific oscillation bands and their interactions (alpha, beta, and gamma) are shown to exhibit these effects. A novel time-domain “phase-autocorrelation function” has been proposed to improve time-frequency resolution, and to better characterize rhythmicity for phase synchronization of oscillations (Myrov et al., 2024).

Attentional control has also been associated with synchronization effects between alpha and beta oscillations, specifically suppression to non-attended visual stimuli in MEG studies (Sacchet et al., 2015). We hypothesize that such push-pull synchronization mechanisms could serve, not only to support attention, but more broadly, to determine selective brain network activations and suppressions, for efficient functional information processing.

We propose that understanding basic wave dynamics is key to understanding information processing in biological networks. Maintaining high resolution temporal and phase information for characterizing stimuli and neural responses is essential for best doing that.

### 4.2 Holography and some holography background

#### 4.2.1 Wave interactions drive holography and its patterns

In 1948, while trying to improve the resolution (reduce blurriness) of electron microscope images, Dennis Gabor suddenly realized that he could possibly use coherent (phase-aligned) electron beams to correct optical aberrations. Based on Thomas Young’s 1801, double-slit light experiments to produce light wave interference patterns, Gabor employed a mercury arc lamp with a narrow-band green filter as a common coherent light source to create an *interference pattern* from two beams, a *reference* beam interacting with an *object* beam reflected off a small transparency inscribed with the names of three wave dynamics pioneers, “Huygens, Young, and Fresnel.” The resulting interference light pattern was recorded on a photographic plate, the first “hologram.” Subsequently when the hologram was illuminated by the same coherent light with which it was produced, a visual image of the object appeared in the same place as where the original physical object had been placed. In contrast to photographic images that only record amplitude information, the hologram records both amplitude and phase information. Furthermore, the information of the interference pattern is distributed throughout the entire holographic image, such that the object can be reconstructed by any piece of the hologram, although resolution decreases as piece sizes are reduced. This unusual characteristic came to be recognized for its striking similarity to the human brain for its ability to recall memories when prompted by one or more memory attributes.

Gabor dubbed his invention “wave-front reconstruction.” Although his invention was quickly acknowledged, and Gabor continued working on it, the reconstructed images remained frustratingly fuzzy, and interest in his methodology waned, until the invention of lasers providing highly coherent light sources, and other improvements.

With the advent of the laser in 1960, a renewed interest in holography and its applications, exploded. Notably amongst these were significant improvements in the three-dimensional holographic methodology, advancing from Gabor’s on-axis holography to off-axis “carrier frequency” holography by Leith and Upatnieks (1962, 1965). Further major advances, relaxing strict coherence requirements, and enabling white-light viewable holograms are attributed to (Denisyuk, 1962; Hoffman et al., 1965; Stroke and Labeyrie, 1966; Benton, 1969; Hartman, 1970) and many others. Gabor was later honored for this discovery with the 1971 Nobel Prize in Physics.

As reviewed by Leith (1976, 1997), some scientists in the late 1940s and 1950s productively applied the same physical principles as used by holography, in their work on wavefield dynamics and interactions, but did not frame it in holographic terms. Lohmann (1956) pursued single side-band radio communications, and Cutrona et al. (1966) worked on improving the optical processing of radar signals (see section “4.3 Radio communications principles generate emergent brain signals”).

#### 4.2.2 Wave interference and holographic processes

Holographic architectures and processes are attractive for many reasons. Their appeal arises from such properties as their self-organizing organic structure, distributed (local and non-local) content representations, computational efficiency, holistic images/entities can be constructed from arbitrary portions, resolution scalability, graceful degradation, and especially, their emergent object reconstruction (e.g., multi-perspective three-dimensional image).

They are created by interference wave patterns, either through analog or digital processes. In optical holograms, a coherent light source is split such that a reference beam is directed toward a recording substrate (e.g., film) where its light waves interact with an object beam, the other portion of the split light source which has bounced off one or more objects placed in its path before interacting with the reference beam. The pattern of the wave interactions of the reference and object beams is recorded on a film substrate and referred to as a hologram. The film is analogous to the self-organized biological memory substrate. In contrast to inert film, biological memory is much more dynamic, and may be modified by subsequent signal interactions.

When the hologram is subsequently viewed using the same coherent light source with which it was generated, a virtual image of the original object itself appears. This holographic image is a product of the reference and object beam wave interactions. It is an emergent property. In contrast to a two-dimensional photograph, the object appears three-dimensionally, and the viewer can view it from multiple angles. Multiple holograms can be even be superimposed on the same film substrate. In contrast to a standard photograph, you can cut up the hologram, and see the entire virtual image through any of the pieces, although the resolution and viewing angles decline as the pieces get smaller.

Digital holography cost-effectively replaces the recording substrate with a digital sensor array (e.g., CCD camera) to capture the interference pattern data (interferogram) which is then analyzed and processed digitally, overcoming many of costs and obstacles of analog holography. Using digital holography, major industries have developed three-dimensional image reconstruction, optical microscopy, medical imaging, industrial measurements and quality control, data storage and mining, military applications, weather forecasting, and more.

#### 4.2.3 Holographic brain analogies

Like holography, neural systems appear to employ wave mechanics and auto-/cross correlations in their operations. Memory, and possibly other biological processes, may be viewed as a self-organized dynamic mesh of competing elements and ensembles. The elements/ensembles themselves are in a state of constant adaptation and modification. Different cell types can respond to different signal attributes. The pattern of ensemble behavior is likely an amalgam of diverse cell states and responses.

In conjunction with previously learned signals and patterns, the neuronal system both auto- and cross-correlates (i.e., compares and contrasts) its background or “reference” state against new/old “object” signal patterns. These new signals can encompass externally or internally generated sensory, motor, cognitive, affective, or other stimuli. Typical familiar contexts can be regarded as reference conditions against which novel stimuli can be detected and compared.

Temporal spike-derived patterns enable the highest resolution for precisely characterizing the spike train signals, -correlating them, synchronizing, and coupling them with others. It is understood that two or more patterns being correlated may co-occur, or be delayed relative to each other (due to different delay lines, recurrence loop dynamics, etc.). Alignment and correlation of two or more such patterns may be triggered automatically through potential threshold triggering and/or designated by discrete bursts or resets. Bursts or resets may act as markers for correlating and coordinating multiple signal pattern interactions and integrations.

Highly correlated patterns can serve to amplify signals (constructive interference). Amplification of signals serves to maintain them as they propagate through the system, analogous to radio signal broadcast repeaters. Alternatively, the combination of antiphasic patterns on either excitatory or inhibitory channels can cancel one another. Interacting signals of different strengths can also provoke a “winner take all,” mechanism where the weaker signal is swamped by a stronger signal, and only the stronger signal survives.

These mechanisms, working in conjunction with recurrent loops, could be the means, by which amplification for learning, reinforcement learning, and habituation is achieved, and by which cancellation mediates variable time course memory loss (e.g., forgetting).

#### 4.2.4 Holographic patterns and reconstructions

Beurle (1956) theorized the wave dynamics by which simple threshold-sensitive neuronal cell ensembles could (1) propagate waves (cf. Keller et al., 2024), (2) show how those waves could be initiated, amplified, attenuated, and adapted, (3) show how their interactions (interference patterns) could be stored, and subsequently retrieved (regenerated) from memory, and (4) show how they could be gated through a series of cellular on/off switches, analogous to attentional focus (Beurle, 1956). Though not referring to holography *per se*, he demonstrated how wave dynamics and interactions are related to holographic principles and properties, and how those mechanisms might operate in living organisms.

The significance of this demonstration was later pointed out by Longuet-Higgins and co-authors: “… one particularly attractive idea emerged from Beurle’s analysis, namely that two different waves spreading across the cortex might together generate an interference pattern from which either wave alone could subsequently regenerate the other… it was explicitly referred to by Van Heerden in a pair of papers (Van Heerden, 1963a,b) which first took seriously the analogy between associative memory and the optical technique of holography” (Beurle, 1956).

Invoking holographic principles, though not holography itself, Roy (1960, 1962) proposed widely distributed sparse representations for robust, associative learning and memory models, “where the stream of information is itself the encoding and decoding device….” Composed of variable delay lines, his “nerve nets” stored autocorrelations to be compared and contrasted to incoming signals, and switched accordingly. Information was conveyed via statistical distributions of variable threshold states between interacting neural ensembles. He proposed recurrent memory replay loops as well, like a “tune running around his head.”

Van Heerden (1970) described the holographic-like reconstructive process, as “the sudden flash of recognition (of a specific person with) absolute certainty (with) extremely reliable and fast information processing in the brain,” a trained recognition process we may regard as “chunking” (see section “4.5 Radio cascade neural mixing model”).

#### 4.2.5 Self-organizing pattern construction and reconstruction

Spatial-temporal signal patterns and their interactions with one another, may be much more fundamental to neuronal processes, at all levels, than is typically conceived. It is the occurrence and recurrence of patterns that (through plasticity and positive/negative reinforcement) generate, and produce our realizations, perceptions, actions, and understanding of the world, underpinning our reactions and responses to it.

The brain can be viewed as a goal/attention-directed, wave pattern interaction machine. Holographic principles and representations are instantiations of that perspective.

Mechanistically, internal/external input signals are waveforms which are encoded (temporally and/or spectrally), propagated and integrated through multiple parallel processing stages of a neuronal network to produce a variety of outputs (perceptions, motor actions, cognition, etc.). These outputs *emerge* naturally as the resulting consequence of the combined effects of multiple competing processes in the organism (e.g., physiological, psychological, motivational, cognitive, etc.).

For example, when a stimulus waveform is detected by one or more modalities, sensory neurons (ensembles) encode the waveform as a series of spike bursts and spike interval sequences. This pattern, and variants of it, can be propagated, projected, and distributed to other regions of the brain, both locally and globally, for further processing and integration.

Early-stage parallel neuronal pattern projections are analogous to the splitting of holographic reference beams. Subsequent processing of these patterns with other patterns, result in new patterns that are analogous to holographic reference and object beams. These neuronal object beam patterns automatically capture multidimensional representations of the object.

If/when these reference beam patterns and object beam patterns subsequently interact with each other, they can interact to form new pattern representations (interference patterns), analogous to holograms. When stored in associative memory, these holographic representations can subsequently be wholly or partially reconstructed (reactivated) when similar (coherent) stimuli are later processed by the neuronal system through memory recall.

Like holograms, partial representations of stored constructs and memories, can be used as object beams, to reconstruct the full original construct or memory representation. This reconstruction incorporates all of that object’s features and attributes. With distributed processing, neuronal pathways coding for partial information (e.g., features like color, sound, timeframe, concept, etc.) can provide multiple entry points for content addressability of that memory. Sufficiently specifying key attributes of any given topic, event, etc., enables unique identification of any entity stored in memory.

Memories have multiple entry points that operate as content addressable access cues or links. Some cues or links are more central or powerful (more distinctive) than others, and provide readier/faster access. Examples include hearing the opening notes of Beethoven’s 5th Symphony, hearing or reading Shakespeare’s “To be or not”…, or seeing a hexagonal red sign post at a traffic intersection. Others may be much more indirect or weaker, requiring the successive activation of multi-synaptic and/or iterative recurrent processes (e.g., delayed recall).

The sensitivity or effectiveness of such cues are directly affected by states of consciousness, attentional selectivity, affective state, and other factors.

#### 4.2.6 Evolution of holographic memory concepts

In his book, *Mechanisms of Memory* (John, 1967), Roy John harkens back to Lashley’s ideas concerning nonlocal, distributed neural representations (Lashley, 1942). Referencing wave dynamics, he cites “nervous networks (developing) a pattern of activity reduplicated throughout an entire functional area by way of excitations, much as the surface of a liquid develops an interference pattern of spreading waves… This means that… the nerves must be sensitized to react in certain combinations, perhaps in complex patterns of reverberatory circuits, reduplicated throughout the area.” He extends existing ideas, discusses multiple mechanisms for brain functions including multimodal integration, synchrony of cortical and thalamic waves, potential roles of neural transients, correlation coefficients related to EEG studies, statistical information processing, etc.

During the mid to late 1960s, a flurry of fertile ideas, theories, and models integrating associative memories, cerebellar and motor learning, with holography were raging and igniting robust debate.

Brindley (1964, 1969) proposed applying the concepts of associative nets, in terms of idealized neuronal structures, using parallel criss-crossed delay lines and modifiable Hebbian synapses, first in the cerebellum for motor control, and then cortically, for learning and sequential recall. Marr (1969) substantially extended Brindley’s work on the cerebellum.

Pribram (1966) quickly embraced relating brain activations to wavefront interference patterns captured in holographic representations. “I have already made the suggestion that arrival patterns in the brain constitute wave fronts which by virtue of interference effects can serve as instantaneous analogue cross correlators… for reconstructions of…holograms (which) have many of the attributes of perceptions….” Although advocating for “temporal coding,” a specific mechanism was not proposed. Pribram (1969) suggested that “transformations that are performed within the input channels can be described in terms of convolutional integrals.” And that these generate “…wave fronts …that set up interference patterns …resembling a hologram.” In his 1971 book *Languages of the Brain* (Pribram, 1971) devoted a whole chapter to holography, its potential neural implementations, and some evidential pros and cons of hypothetical holographic neural mechanisms.

Having written his Ph.D. dissertation (Westlake, 1968) on the theoretical application of neural holographic processes to brain functions, Westlake then directly related his work to corresponding models by Hebb, Pribram, Beurle, and others, to explain how holographic properties (e.g., visual object coding/reconstruction, distributed associative memory, and recurrent loops) could be instantiated with neural pulse waveforms (Westlake, 1970). Along similar lines (Cavanaugh, 1975) proposed a holographic mechanism based on wave interference patterns.

#### 4.2.7 Holographic associative memory models

A brief paper by Longuet-Higgins (1968a) titled “A Holographic Model of Temporal Recall” quickly attracted attention. He proposed a temporal analog to optical holography, using an adaptive filter bank of narrow band pass resonators (filters) coupled to the input signal for recording the temporal signal, and to which when a subsequent response is fed back into the filter bank, the original signal will be regenerated. A series of discussion papers ensued. Another paper that year, “Non-local Storage of Temporal Information” (Longuet-Higgins, 1968b), presented a Fourier-based frequency-domain theory of a temporal holograph, the “holophone,” which could function as a content-addressable memory and reconstruct full temporal sequences from partial sequences.

In response, Chopping (1968) suggested an alternative based on using unique complex patterns determined by the frequency of pulses (spikes), also avoiding the requirement for coherence in filter bank resonances. He proposed an implementation using widely distributed, highly redundant, self-selected, adaptive cells.

In addressing Longuet-Higgins quest for a temporal holographic model, Gabor (1968) weighed in with his own holographic memory models. He proposed storing memories in the form of truncated autocorrelograms, and implementing recall using truncated convolutions, in order to recall an entire temporal sequence from a memory fragment. Further refining his models, he demonstrated that holographic recording and recall functions could be performed with only three operations; shift, multiplication, and summation (Gabor, 1969). Note that in the time-domain a shift corresponds to a delay. He further asserted that, in principle, McCulloch-Pitts neurons ought to be able to implement these operations, though leaving the details to others.

Questioning the biological plausibility of the brain Fourier analyzing incoming signals, Willshaw et al. (1969) suggested that correlations or convolutions of signals might be used as an alternative to sufficiently imitate the behavior of a Fourier holograph.

While acknowledging that holographic functions of the brain were in an early state, Van Heerden (1970) understood and asserted that the foundation was clear. “This foundation is information theory, the same theory which is used in radio, television, radar, and photography. In information theory, recognizing, or speaking of the quantitative degree of two things being alike, is described by the correlation function of two time functions, or two images…The computation of the correlation function can be described mathematically as a (matched) filtering operation…The fact that the hologram performs this filtering function…is due to the fact…that a propagating wave field carries out automatically this laborious computation demanded by the theory.”

Longuet-Higgins et al. (1970), observed the “that the power spectrum and the autocorrelation of a pattern are Fourier transforms of each other, so that to record the one, is equivalent, in terms of information, to storing the other.” Furthermore, they suggested “storing directly the correlations between or within various input signals without the need for Fourier analyzing the signals…” (Longuet-Higgins et al., 1970). By “storing the correlations directly rather than as power spectra…the associative net resembles the holograph,” but “differs from the holograph in not requiring any transformation of the input and output signals, or any coherent source of excitation for this purpose, in employing threshold elements to get rid of unwanted noise in the recall, and in using on-off switches….” The holographic theory predicts the feasibility of achieving a content-addressable associative memory. One of his last papers (Longuet-Higgins, 1989) outlined how holographic memory could be implemented in the time domain using temporal correlations and convolutions of pulse trains.

Drawing on the work of Gabor (1968), Longuet-Higgins (1968a), and Barsellino and Poggio (1972) used convolutions and correlations to propose a holographic mathematical model of temporal memory analogous to Reichardt’s insect optomotor response theory (Reichardt, 1957; Reichardt, 1961; Reichardt and Poggio, 1976). Later Kohonen (1987) incorporated delayed feedback as a key feature in his autoassociative model for the temporal coding and recall of sequences.

Further extending holographic models in his book *Holographic Reduced Representation*, Plate (2003) described his methodology for using circular convolutions for efficiently associating and binding distributed complex composite representations, and applied these to associative sequential memories, learning, chunking, and more. Nickel et al. (2016) proposed using circular correlations for holographic embeddings of knowledge graphs. See Hinton and Anderson (1981) for a review of alternative associative memory models.

Characterized by temporal signals and processes, wave interference patterns may generate holographic-like patterns and representations. These may include memory traces of variable duration/persistence (short- and long-term memory), which can be modified to create new patterns and representations, as well as reactivated by pattern similarity matching operations.

The holographic patterns are more general than memory traces *per se*. These patterns and representations could potentially be propagated and communicated in a manner analogous to radio broadcast operations. In contrast to radio broadcasts though, neural signal patterns may be modified and integrated with other signal patterns along the broadcast route.

### 4.3 Radio communications principles generate emergent brain signals

Radio communications typically deal with broadcasting high frequency transmissions through space. Driven by wave dynamics, their principles support a host of applications from garage door openers to satellite communications, radios, TV, long distance telephone echo cancellation, etc. Echolocation is used for radar, sonar, seismological exploration, etc. as well as by animals; bats, whales, dolphins, etc. Despite their many differences, radio communication systems also bear some striking similarities to brains and their neural communications. These principles, applied at physiological frequencies, may be useful for understanding some specific brain mechanisms, especially, with respect to coordinating different oscillation frequency bands and their interactions in the course of processing, propagating, and communicating information.

#### 4.3.1 Superheterodyne radio: single-sideband carrier suppressed

**FIGURE 3:**
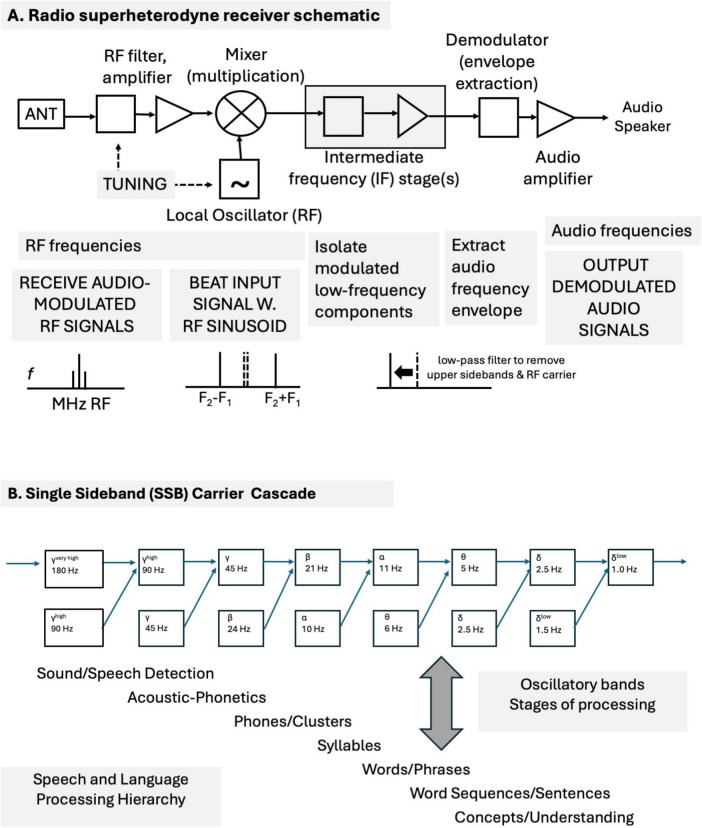
**(A)** Functional signal processing schematic for an AM radio receiver (from Cariani and Baker, 2022). The shaded box with the IF stages contains sequential mixing operations that are analogous to an oscillatory cascade. **(B)** Oscillatory cascade. Emergent frequency band oscillations and approximate corresponding stages of speech and language processing. Carrier cascade of single sideband (F2 – F1) oscillatory frequencies. For graphical clarity, additional putative loops (self, level skipping forward and backward) are omitted.

Audio radio broadcasts, first transmitted in the early 1900s, rapidly evolved to become a household sensation by the 1920s. Basically, transmitters connected to antennas and receivers are able to communicate signals with which they share common signal characteristics. Transmitters engage signal modulations to propagate waveform information over designated channels from one place to another, where it is decoded (see Figure 3).

An Amplitude Modulated (AM) radio architecture typically incorporates a superheterodyne circuit that mixes (multiplies) two signal frequencies F1 and F2 to produce new frequencies. The process of “mixing” is a nonlinear multiplicative operation which results in two new sideband signals, at their F1 + F2 sum frequencies, and their F2 − F1 difference frequencies. Lower amplitude harmonics of these may also be generated. In this fashion, modulated signals can be frequency shifted as needed.

After broadcast transmission, propagation, and reception, the modulation signal is then demodulated and the original signal reconstructed (recovered) for output (playback) at the target site. The original signal information is carried by the sidebands, but *not* in the carrier itself. The two sidebands are redundant in so far as each contains a representation of the information in the original signal. Typically, the upper sideband is eliminated by a low-pass filter for efficiency and power savings, so that only the new F2 − F1 sideband is transmitted.

A succession of these stages can be multiplexed, processed, and cascaded for flexible signal integration and propagation. A cascade occurs when the output of one system stage serves as the input for another system stage. When the carrier itself is suppressed, the operation is referred to as single-sideband carrier suppressed (SSBCS), an especially efficient and widely used transmission mode (Silver, 2012). A reduced–carrier single sideband mode preserving some of the original carrier signal, can also be used to facilitate demodulation.

### 4.4 Brain wave oscillations as potential signal carriers

In researching human electrical brain activity, Hans Berger first observed periodic alpha oscillations (8–10 Hz), and subsequently beta oscillations (15–30 Hz). In 1924, he developed a recording technique using non-invasive scalp electrodes, dubbed electroencephalography (EEG), actively used today. He published his work in 1929, and instigated a vast field of brain research, research tools (e.g., MEG, ECoG, and intracranial electrode arrays), diagnostic and therapeutic applications, etc. (Berger, 1929).

A number of endogenous brain oscillations have since been observed and explored in different frequency bands; alpha, beta, delta, gamma, mu, theta, ripples, spindles, etc. and narrower band ranges of these; e.g., multiple gammas, multiple betas, multiple deltas, etc. (Buzsaki and Draguhn, 2004; Buzsáki, 2006; Buzsaki and Watson, 2012; Han et al., 2021; Garrett et al., 2024). Periodic activities, typically described as oscillations in these frequency ranges are associated with diverse feed forward, feedback, memory access, attention, excitatory, inhibitory, and other processes.

Commonly observed aperiodic spiking signals, such as spike bursts or volleys, or other aperiodic events, even single spikes, may mark new stimuli (e.g., onsets and offsets), phase resets, signal discontinuities, state changes (e.g., shifts to different frequency bands or processing stages, integration events), initiation of new signal paths and processes (e.g., plasticity), etc. Vinck et al. (2024) has reviewed possible roles of periodic oscillatory vs. aperiodic transient signals in the context of hierarchical predictive processing of bottom-up gamma and top-down alpha/beta rhythm interactions. Xie et al. (2024) has demonstrated that categorization information can be embedded in spike sequences of population transient spike bursts, separable from averaged spike rates and latencies from stimulus onset.

Despite extensive research, the fundamental nature, roles, and relationships of periodic oscillations and aperiodic spike bursts, etc., are still a subject of uncertainty and active debate.

Neural systems are likely to process incoming signals in a consistent manner, irrespective of whether or not the incoming signals are periodic or aperiodic. They seamlessly must accommodate diverse signal to mediate, integrate, and control local and global information processing throughout the body. Physical wave dynamics and interactions drive these signal-centric processes.

Understanding oscillation interactions appears to be one means of doing this, such as gamma oscillations, typically associated with bottom-up sensory input, interacting with beta top-down predictive and/or inhibitory activity, etc. The interacting signals in these different bands, combine, couple, and mix with each other. Such interactions can act to amplify, suppress, modulate, tune, and coordinate a broad range of neural signals. Massively parallel crosslinked network channels can provide redundancy, sharpen SNR, amplify and attenuate signals, detect and correct errors, etc. for robust signal processing and propagation.

### 4.5 Radio cascade neural mixing model

Oscillation co-occurrence and coupling of diverse frequency bands have been broadly observed across the brain. Co-occurring phase-locked ripple oscillations (∼90 Hz) are observed to synchronize across widely separated brain regions, during both sleep and waking states (Lestienne, 2001; Dickey et al., 2024; Verzhbinsky et al., 2024). These can increase during reading and semantic decisions (Garrett et al., 2024), and may be preceded by very high gamma bursts (120–190 Hz). Increasingly, studies have invoked basic frequency signal mixing principles, as a key mechanism, to explain oscillation signal band interactions serving various functions (Ahissar et al., 1997; Ahrens et al., 2002; Haufler and Pare, 2019; Dickey et al., 2022; Staresina et al., 2023; Staresina, 2024; Tye et al., 2024), including demonstrating signal mixing without using band-pass filters (Kleinfeld and Mehta, 2006). An extensive analysis of diverse signal mixing of exogenous and endogenous frequencies has been conducted in rodent brains. A subsequent study of human EEG signals identified brain regions where different oscillation bands predominate and demonstrated with which other bands they preferentially mix in the context of an attentional feature-matching task (Luff et al., 2024).

Applying radio communications theory, we hypothesize that these different oscillation frequency bands could *broadly operate* as *signal carriers*, within the brain and neural system. If this is the case, then specific oscillation bands could be more closely associated with different stages or types of information processing and multimodality integration, for speech, image, motor action, cognition, homeostatic functions (blood pressure, orthostatic stability), etc. The constant generation of new signal channels with new frequency, phase, and amplitude characteristics could enable the progressive flow of phase-sensitive, channel selective synchronous coupling with other neurons, networks, and sub-networks (e.g., lexical networks coupling with semantic networks). Commensurably, as new frequencies are being emergently generated, the mixing frequencies from which they are generated, will be reduced or eliminated. Recurrent activations could maintain and reinforce information while it remains relevant, as well as attenuate or suppress it when it is not.

In support of these ideas, we extend a radio communications-based model, previously introduced (Cariani and Baker, 2022). In radio terms, this specific design is referred to as “single side-band” (SSB) or “single side-band suppressed carrier” (SSBSC). It is a superheterodyne circuit typically applied to AM radio where one of the two sidebands, and optionally, the carrier wave itself, are beneficially suppressed to conserve power and bandwidth. In this model, *neural oscillation frequency bands are the carrier waves* corresponding to AM radio intermediate frequencies. Each stage is characterized by a different oscillation frequency carrier (Figure 3B).

When a carrier frequency is mixed (multiplied) with another frequency (the local oscillator), two new frequencies (F2 + F1 and F2 − F1) are automatically generated. In the heterodyning process, only the lower F2 − F1 frequency is transmitted as the new carrier frequency for the modulated signal. The summed F2 + F1 frequency is typically eliminated by low-pass filtering.

Thus, the frequency of each carrier, is *emergently* determined by the difference frequency of the two previous oscillation bands (carrier and local oscillator). Since there is a succession of *difference* frequencies being generated and propagated, later stages necessarily produce lower frequency oscillation carriers than previous stages. Successive stages of these intermediate frequencies are sequenced in a “cascade,” where the output of one stage feeds the input of the next stage.

As a theoretical illustration, consider a hierarchy of neural processing states for speech/language processing, approximated as a progression and integration of sensory to cognitive processing stages; sound detection, speech detection/activation, speech acoustics, phones, syllables, words, phrases, sentences, and concepts/understanding. Suppose that each of these speech/language stages, is represented by the center frequency (mean) of the frequency band with which it is typically associated, e.g., for example, 180 Hz (very high gamma), 90 Hz (high gamma, ripples), 45 Hz (gamma), 24 Hz (beta), 10 Hz (alpha), 6 Hz (theta), 2.5 Hz (delta), and 1.5 Hz (low delta).

When each of these frequencies is mixed (multiplied) with the previous carrier frequency, a new carrier frequency is produced. For example, multiplying a 180 Hz (very high gamma) signal with a 90 Hz (high gamma) signal produces a 90 Hz carrier, a 90 Hz (high gamma) carrier multiplied with a 45 Hz (gamma) produces a 45 Hz (gamma) carrier, a 45 Hz (gamma) signal multiplied with a 24 Hz (beta) oscillation produces a 21 Hz (beta) carrier; a 21 Hz (beta) carrier multiplied by a 10 Hz (alpha) oscillation produces an 11 Hz (alpha) carrier, which mixes with a 6 Hz (theta) oscillation to produce a 5 Hz (theta) carrier, mixing with a 2.5 (delta) oscillation to produce a 2.5 (delta) carrier, mixing with a 1.5 Hz (low delta) oscillation, to produce a 1 Hz (low delta) carrier. This sequence of *emergently* generated carrier frequencies; 90, 45, 21, 11, 5, 3, and 1 Hz, closely recapitulates the sequence of the commonly observed oscillation frequencies, as noted above; i.e., 90 Hz (high gamma), 45 Hz (gamma), 24 Hz (beta), 10 Hz (alpha), 6 Hz (theta), 2.5 Hz (delta), and 1.5 Hz (low delta), approximately reducing the dimensionality of the original frequencies, by a factor of 90–180X.

Here we observe a trend of higher frequency carriers corresponding to lower-level processing stages, and lower frequency carriers corresponding to later higher-level processing stages, indicative of hierarchical consolidation (e.g., sequence of sound/speech detection and phone acoustics consolidated to words and to conceptual understanding). Not surprisingly, the initial, lowest level stages for sound/speech detection and acoustics, are the fastest. The mechanism proposed here generates oscillatory sequences similar to those of Kramer (2022) in which neighboring neural frequency band relationships are related by the (Fibonacci sequence derived) “golden ratio” (∼1.618).

The cascade example above in Figure 3B demonstrates a hierarchical sequence corresponding to characteristic speech and language stages. Most of the neuronal mixing literature to date, has dealt with only one or two mixing stages in localized areas. We hypothesize that longer sequences of mixed oscillatory bands might well exist in different brain regions and at different times. The above cascade model predicts a specific sequence of oscillatory bands for speech and language processing, such that it could be tested using data from neurophysiological recordings.

Note that when two frequencies (F1 and F2) mix to produce (generate) a new frequency (F2 − F1), the frequencies F1 and F2 themselves, are abolished in the process. In actual practice, with electronic SSBCS circuits, remnants of the generating frequencies may remain, as well as low amplitude frequency harmonics. In neural systems, the rapid creation and abolition of observed oscillations could explain transient durations.

Given the large frequency range of individual neural oscillation bands, often spanning an octave or more; e.g., gamma (∼40–180 Hz), beta (∼13–30 Hz), and delta (∼0.5–3.5 Hz), it is hardly surprising that there are meaningful distinctive narrower bands reported within these. Furthermore, the F2 − F1 generation of new carrier frequencies, can easily result in the *same* carrier frequency, e.g., alphas, betas, *simultaneously* serving comparable or different functions with different functional connectivities, in different regions (sub networks) of the brain. If so, that might partially explain the diverse roles (sensory, motor, cognitive, attention, memory, etc.) commonly observed for multiple distributed alphas (Klimesch, 2012) and betas (Weiss and Mueller, 2012; Lundqvist et al., 2020).

The distinctions between different frequency carriers enable rapid selection of allowed, and rejection of disallowed cross-frequency channel synchronizations and couplings. This general signal mixing principle is broadly applicable across different processing stage levels, not just neighboring levels. For example, this principle applies to interactions of afferent and efferent signals, e.g., bottom-up, top-down information combinations and integrations.

This mechanism enables the processing, adaptation, and propagation of information across and within multiple channels and networks, locally and globally across the cortex. It is likely that most transitions are to elements *within* a given level, e.g., between alternative syllables, between alternative words; that is to whichever level element is most likely, i.e., best predicted by stimuli (feedforward) and historical context (feedback). The elements within any given level consist of pretrained (tuned) adaptive cell assemblies. For example, in a speech processing hierarchy, such pretrained cell assemblies can correspond to separate levels for phones, syllables, words, syntactic relations, concepts, etc.

In this model, we hypothesize that there are necessarily backward loops from higher processing levels to lower processing levels (e.g., theta to beta and delta to theta) to enable reprocessing of ambiguous or low confidence signal interpretations. Reprocessing can take place at all levels; e.g., acoustic uncertainty (was the word “affect” or “effect,” “fine” or “find”), semantic (word sense) uncertainty (“overlook,” “rent,” “back up,” and “remains”), etc. Confidence (certainty) levels are determined by the relative likelihoods of bottom up (feed forward) signal processing and top down (feedback) predictions. Reprocessing triggers occur when the confidence disparity of the most likely candidate(s) falls below a threshold, or confidence discrimination between top candidates falls below a threshold. Likely neural signals reflecting such triggers for reprocessing include the N400 (Kutas and Hillyard, 1980, 1984; Wang et al., 2012) and P600 (Osterhout and Holcomb, 1992; Schneider and Maguire, 2018).

Note that in automatically generating text, Artificial Neural Networks (ANNs) typically emulate this kind of behavior by predicting the next most likely word based on Large Language Models (LLMs) using n-gram statistics, weights, and thresholds to choose paths through their graphical networks.

### 4.6 Multilevel processing and chunking

When there are larger trained cell assemblies, encompassing a higher level of processing elements e.g., words, concepts, etc., the direct pattern detection and recognition of these chunks enables much more rapid information processing than working lockstep up from lower levels in the hierarchy. However, when there is a high level of ambiguity or uncertainty (e.g., did she say “nice peach” or “nice speech”), then lower levels of the hierarchy must be analyzed. Use of higher-level chunks is gated by thresholds, and confidence level differences between competing chunks. Max confidence determines choice selection.

Basically, as long as there is a close fit between a top-down prediction and a bottom-up confidence score, processing can advance. But if there a disparity (simple difference) between two (or more) measures, that exceeds a threshold, and/or confidence levels in bottom-up signals are too low, then additional analysis or re-processing, and the associated processing time, is required.

A similar very rapid hierarchical categorization or chunking process is described by Serre et al. (2007) for high performance visual object detection and recognition by macaques and humans. The methodology reflects a hypothesis for the ventral stream generating a “large vocabulary of shape-tuned units” (high level visual chunks) derived from a succession of processing stages between primary visual cortex (V1) and the prefrontal cortex (PFC). A similar hierarchical feature clustering, aggregation, and rapid recognition process also appears evident for gisting in visual scene analysis (Oliva and Torralba, 2006). Gestalt recognition and other invariance properties may be served by chunking mechanisms. From this and other evidence, we view chunking as a very powerful adaptive processing and learning methodology.

In the field of automatic speech recognition, substantial speed-ups in processing are achieved using a chunking like technique known as “rapid match” or “fast match” (Gillick and Roth, 1990). Beginning word acoustic clusters are categorized and trained for all vocabulary words. The search space for recognizing subsequent speech is then sharply pruned by restricting search only to those word paths containing similar start clusters. As a consequence, the speech recognition process often reliably recognizes the word being spoken well before the speaker has finished speaking the entire word!

### 4.7 Recommendations for observing precision timing

Because most studies to date are based on averaged, frequency-domain processing perspectives, rather than employing or incorporating analytical techniques with high temporal resolution, there is necessarily ambiguity in currently testing these studies for more direct correspondence, or lack thereof, to our ideas and theory. We are eager to have our hypotheses tested.

Reporting frequency results with observations measured in exact hertz with variance, standard deviations, etc., rather than broad frequency bands would clearly help (Hadjipapas et al., 2022). It would enable better characterization and understanding of neural activity, especially for signal integrations and interactions. Some studies already do this routinely, but many do not. Despite these disparities, it is informative to examine current empirical studies to assess areas and timeframes of activity correspondence across the brain (Luff et al., 2024; Lundqvist et al., 2024). Spike-interval based measures and patterns (e.g., phase-locking analysis for spike trains and bursts) can provide higher temporal resolution, which could potentially prove very valuable.

Many physiological studies have generated empirical evidence, typically in specialized areas and domains, supportive of these kinds of mechanisms, and the broad framework we are proposing. Much more testing from a temporal perspective, will be required to confirm or reject their predictive power, and extent of their applicability.

## 5 Discussion – testing the time-domain theory

Many of the mechanisms of the neural system can be parsimoniously described in the time domain by basic laws of physics and wave dynamics, using correlations, convolutions, and delay lines.

Evidence for these is widely observed in neuroscience research, and has been for decades.

Recent research findings increasingly support the need for high temporal, amplitude, and phase resolution, as well as for mechanisms (e.g., delay line integrations, phase-sensitive coupling dependent on these).

A temporal perspective can facilitate a highly integrated, consistent, and holistic framework for understanding the brain operations from which diverse significant neural functions (e.g., perception, motor action, cognition, etc.) may simply *emerge*!

The theory proposes that signals, signal patterns, and their dynamics are central to shaping their interactions, brain activations, and the brain architecture itself. It focuses on the emergent nature of pattern creation and reconstruction, arising from the laws of nature (rather than elaborate algorithmic constructions) applied to signal-centric codes and patterns, and processed by plausible brain mechanisms.

This signal-centric brain theory is demonstrated using holographic principles and heterodyned radio communications theory. Spatial-temporal signal patterns, and sequences of these, may be coded, modulated, modified, stored, retrieved, integrated, demodulated, etc. for propagation and transmission through widely distributed, networks of parallelized cell ensembles to support many brain functions and behaviors. An evidence-based sketch of how this could work, and an illustrative model based on putative oscillation interactions are offered.

We do not contend that we have “figured it all out” or anything of the sort. We warmly encourage further research in this direction and propose some specific recommendations to facilitate it. There are significant gaps in putting it all together. Experimental evidence is required to test these hypotheses, and their extent. That will require that neural data collections first take care to preserve the temporal resolution inherent in the signals themselves, by adequate sampling, and avoiding destructive filtering in subsequent processing and analysis. Preserving temporal resolution means avoiding automatically imposing windows with their inherent time-frequency resolution tradeoffs. It means not averaging signals across variable populations or data during non-steady states. These conventional practices, though computationally convenient and supported by popular tools, often destroy critical information, especially temporal information in biologically meaningful complex signals. More detailed investigation needs to be undertaken to study interspike intervals for coding and processing, for single neurons, ensembles, and populations. Critically, a willingness to try out new practices will be required to pursue this perspective. Breaking with convention is never easy! Some new tools would be helpful. These will definitely take some work and development

Preserving high temporal (spike timing) resolution is fundamental for characterizing, coding, and explaining the brain signals and signal processing which support brain mechanisms, functions, and behaviors. More and more evidence confirms that many brain signals and interactions (spike bursts, phase-locking, phase-amplitude coupling, etc.), operate on much more rapid (millisecond and sub-millisecond) timescales than are captured by traditional spectral power windows (tens to hundreds of milliseconds). Like telescopes and microscopes, better temporal resolution will allow us to see and understand much more.

A number of general suggestions for empirical investigations were listed in our last paper (Cariani and Baker, 2022).

To recapitulate, the main assertion of the time domain theory presented here is that the neural codes used to realize the major informational functions listed in section “2 Basic brain functionalities (what is to be explained)” and Table 1 may consist of temporal patterns of spikes rather than rate-place patterns. This includes the neural coding of sensory, perceptual cognitive, affective, conative, executive, and motoric distinctions. The theory holds that segmentation and binding processes are subserved by temporal correlation-based mechanisms. Multidimensional vectorial representations consist of co-occurring temporal patterns associated with individual attributes of objects, events, situations, and procedures. Memory traces may consist of complex temporal patterns of spikes that encode the attributes of events as well as their timings and sequences.

Testing the validity of these assertions entails solving the neural coding problem for each of these domains. This involves looking for the postulated temporal spike codes with sufficient temporal resolution to determine whether they can accurately predict with precision and robustness the corresponding informational functions. The coding differences need to be shown to be causal for the functions they are postulated to support.

Search for complex and multiplexed temporal codes will require analysis of spike patterns that possibly occur across spike trains of different neurons (see section “3.1.6 Finding temporal codes in neural activity patterns”). Electrical, magnetic, optical, and acoustic stimulation in different temporal patterns may also constitute evidence for the efficacy of temporal patterns in evoking different functional brain states.

We further posit (see section “4 Time-domain waveforms, signals, and systems: common signal operations, holography, radio communications, and the brain”) that time-domain representations and operations can be implemented using temporal correlations, time-domain holographic-like processing, wave interference patterns, and/or oscillatory cascades. Mathematical analysis and signal-processing simulations would be useful for demonstrating the feasibility of these operations. Extending experiments along the lines of Luff et al. (2024), more extensive evidence of nonlinear multiplicative and thresholding operations, especially in the context of oscillatory cascades (see section “4.5 Radio cascade neural mixing model”) could be carried out. Frequency-tagged stimuli could prove useful for such experiments.

To the best of our knowledge, our model is novel in applying signal processing principles to relate oscillatory band interactions predictively across the range of commonly observed neural oscillation bands. Empirical testing will be required to further refine, support, or refute our hypotheses explicitly. Ways to do that would be to inject additional oscillatory stimuli and observe interactions, both for generating and/or disturbing endogenous oscillations under different task paradigms (Takahashi et al., 2024), to apply external stimuli to antiphasically abolish endogenous oscillations, and to pharmacologically disrupt known communication pathways. The logistics for performing such research, especially simultaneous recordings at multiple sites, precision temporal recordings, etc. will prove challenging but are not perceived to be beyond the state-of-the-art.

## 6 Conclusion

A time-domain, signal-centric theory of brain function is possible and plausible. Here we propose a set of principles for temporal coding and processing that rely on the dynamics of signal interactions. Relatively simple time-domain mechanisms are proposed. These are integrated and coordinated in a coherent functional organization that can support widely observed complex brain functions and behaviors. These principles and mechanisms operate at multiple levels, both locally and globally.

Starting with basic codes and operations (e.g., temporal spike codes, correlational processing, signal synchronization, signal mixing principles, and spreading activations), certain functions and processes (e.g., spatiotemporal holographic object and sequence reconstruction, content-addressable memory, and oscillation interactions for propagating information selectively), may efficiently and elegantly, simply *emerge*.

We believe this temporal perspective will prove useful in formulating a coherent consolidated framework for understanding the brain. There remain many open questions that are amenable to empirical testing, and which we hope, with others, to investigate more fully in the future.

## Data Availability

The original contributions presented in this study are included in this article/supplementary material, further inquiries can be directed to the corresponding author.

## Footnotes

1 Cariani, P., and Baker, J. M. (2025). Survey of temporal coding in sensory systems. *Front. Comp. Neurosci.* (in review).
